# Investigation of hydrated channels and proton pathways in a high-resolution cryo-EM structure of mammalian complex I

**DOI:** 10.1126/sciadv.adi1359

**Published:** 2023-08-02

**Authors:** Daniel N. Grba, Injae Chung, Hannah R. Bridges, Ahmed-Noor A. Agip, Judy Hirst

**Affiliations:** The Medical Research Council Mitochondrial Biology Unit, University of Cambridge, Keith Peters Building, Cambridge Biomedical Campus, Hills Road, Cambridge CB2 0XY, UK.

## Abstract

Respiratory complex I, a key enzyme in mammalian metabolism, captures the energy released by reduction of ubiquinone by NADH to drive protons across the inner mitochondrial membrane, generating the proton-motive force for ATP synthesis. Despite remarkable advances in structural knowledge of this complicated membrane-bound enzyme, its mechanism of catalysis remains controversial. In particular, how ubiquinone reduction is coupled to proton pumping and the pathways and mechanisms of proton translocation are contested. We present a 2.4-Å resolution cryo-EM structure of complex I from mouse heart mitochondria in the closed, active (ready-to-go) resting state, with 2945 water molecules modeled. By analyzing the networks of charged and polar residues and water molecules present, we evaluate candidate pathways for proton transfer through the enzyme, for the chemical protons for ubiquinone reduction, and for the protons transported across the membrane. Last, we compare our data to the predictions of extant mechanistic models, and identify key questions to answer in future work to test them.

## Teaser

Extensive hydration networks highlight key regions for the redox-coupled proton-translocating mechanism of mammalian complex I.

## INTRODUCTION

Respiratory complex I [NADH (reduced form of nicotinamide adenine dinucleotide):ubiquinone oxidoreductase] is a key enzyme in mammalian metabolism and a redox-driven proton pump central to oxidative phosphorylation in mitochondria and aerobic bacteria. It catalyzes NADH oxidation and ubiquinone reduction, regenerating nicotinamide adenine dinucleotide (NAD^+^) to sustain oxidation of carbohydrates and fats by the tricarboxylic acid cycle and β-oxidation and feeding electrons into the electron transport chain for O_2_ reduction. It also captures and “couples” the free energy liberated by NADH:ubiquinone oxidoreduction to transport four protons across an energy-transducing membrane, contributing to the proton-motive force (Δ*p*) that is essential for adenosine triphosphate synthesis and transmembrane transport. The convergence in complex I of NAD^+^/NADH redox homeostasis, respiration, and oxidative phosphorylation defines it as a vital hub of energy conversion, so its dysfunctions lead to many neuromuscular and metabolic diseases (*1*–*3*).

**Fig. 1.**
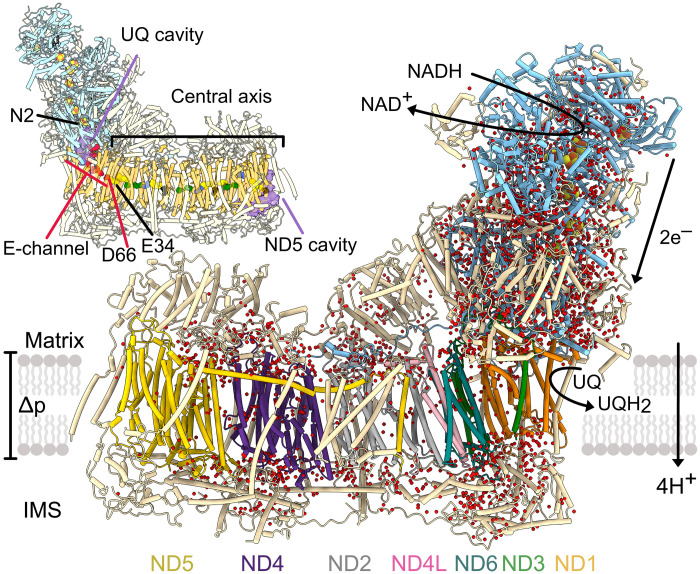
Water molecules and cavities in the structure of the active resting state of mouse complex I. A total of 2945 modeled water molecules are shown as red spheres. All subunits are displayed in cartoon representation with the 7 hydrophilic core subunits in blue and the 31 supernumerary subunits in wheat. The seven transmembrane core subunits are colored as labeled (ND5, yellow; ND4, purple; ND2, gray; ND4L, pink; ND6, teal; ND3, green; ND1, orange). Electrons are transferred from NADH to the flavin mononucleotide in the hydrophilic domain and then sequentially down a series of FeS clusters (orange–yellow spheres). The terminal FeS cluster N2, situated above the membrane, reduces the membrane-bound ubiquinone-10 (UQ) substrate to ubiquinol (UQH_2_) in a binding channel at the interface of the hydrophilic and membrane domains. The redox reaction drives the translocation of four protons through the membrane domain, generating Δ*p*. The inset highlights the ubiquinone-binding and ND5 cavities (purple), plus the conserved residues of the E-channel (red, final residue ND3-D66) and central axis (first residue ND4L-E34) with the ND2, ND4, and ND5 TMH5-7 Glu–Lys pairs in green, TMH8-His/Lys in blue, TMH12-Glu/Lys in black, and other polar residues in yellow.

Mammalian complex I is a 1-MDa complex in the inner mitochondrial membrane that contains 14 core subunits, conserved from bacteria to humans, plus a mammalian-specific cohort of 31 supernumerary subunits required for enzyme stability, regulation, or assembly (Fig. 1) (*4*–*7*). The core subunits house the catalytic machinery, the key features and framework of which have been elucidated by structural analyses (*6*, *8*–*18*). NADH is oxidized by a flavin mononucleotide at the top of the hydrophilic domain in the matrix; then, the two electrons released traverse a series of iron–sulfur (FeS) clusters to the terminal cluster (N2, just above the hydrophilic–membrane domain interface) to reduce ubiquinone (Fig. 1). The hydrophobic ubiquinone (typically ubiquinone-9 or ubiquinone-10, here referred to also as UQ10) enters from the membrane, migrating along a long narrow channel to position its redox-active headgroup adjacent to N2, ready for ubiquinone reduction to trigger proton translocation in the 200-Å-long membrane domain. The ubiquinone-binding site is intimately connected with the E-channel, a hydrated network of residues dominated by glutamate (E) residues, that leads down into the membrane domain through subunit ND1 and ends at ND3-D66 (Fig. 1). ND3-D66 is ~12 Å from ND4L-E34, the first residue of the central axis, a chain of charged residues running longitudinally along the membrane plane. Between them, the status of transmembrane helix 3 (TMH3) in subunit ND6 (ND6-TMH3) determines the connectivity between ND3-D66 (the E-channel) and ND4L-E34 (the central axis) (*8*, *10*, *11*, *13*, *17*, *19*, *20*). In “active” or “closed” states of the enzyme, in which the ubiquinone-binding site is fully ordered, ND6-TMH3 is α-helical: The region around it is hydrated, and the E-channel is connected to the central axis by a hydrogen-bonding network. In contrast, in “deactive” or “open” states, in which loops that form the ubiquinone-binding site appear poorly ordered, ND6-TMH3 contains a π-bulge: Bulky residues rotated around the helical axis displace water molecules, dehydrating the region and breaking the connection. The central axis (Fig. 1) connects subunit ND4L to three antiporter-like subunits—ND2, ND4, and ND5—that are essential for proton pumping. Key conserved features of the central axis in these subunits (*21*–*28*) are a glutamate–lysine ion-pair (TMH5–TMH7) where the axis enters the subunit, a lysine or histidine on TMH8, and a lysine or glutamate on TMH12 where the central axis leaves the subunit (or terminates).

Despite extensive structural, functional, and computational work, both the coupling mechanism between ubiquinone reduction and proton pumping in complex I and the pathways and mechanisms of proton translocation remain under debate. Water molecules are key to these pathways and mechanisms, but until recently, it has only been possible to investigate them by computationally hydrating medium-resolution structures. Now, with the ever-improving resolution of single-particle cryo–electron microscopy (cryo-EM), the densities of ordered water molecules have been observed and modeled in the enzymes from *Yarrowia lipolytica* (*11*, *17*), *Ovis aries* (sheep) (*13*), *Sus scrofa* (pig) (*12*), *Bos taurus* (cow) (*10*), *Escherichia coli* (*16*), *Arabidopsis thaliana* (*14*), and *Chaetomium thermophilum* (*29*).

Here, we present a 2.4-Å global resolution map of complex I from mouse heart mitochondria with ~3000 water molecules modeled. Our structure has a fully configured ubiquinone-binding site that is sealed from the matrix and an α-helical ND6-TMH3, so it is in the closed mammalian active resting state (*6*, *8*, *10*, *30*, *31*). As a result, we do not discuss the open or deactive resting states of the mammalian complex. Although determining whether the enzyme “opens and closes” during catalysis is key to understanding its mechanism, we have recently reviewed this topic (*31*) and focus here on structural analyses of charged and polar residues and water molecules to evaluate potential proton transfer pathways and protonation states. We compare our results to the predictions of extant mechanistic models and highlight key questions to answer in future work to test them.

## RESULTS

### Overview of the structure of the active state of mouse complex I

Complex I was purified from mouse heart mitochondria (*8*), frozen onto PEGylated gold grids (*30*, *32*, *33*), and imaged on a Titan Krios microscope. A total of 109,866 particle images were processed and refined into a final map of 2.4-Å global resolution with good orientation distribution and consistent local resolution (table S1 and figs. S1 and S2A). Both the global enzyme conformation and local conformations of key distinguishing features [ND6-TMH3, ND1-TMH4, loops in ND1, ND3, NDUFS2, and NDUFS7 (fig. S3A)] define the structure as in the active resting state (*6*, *8*, *10*, *30*, *31*).

The resolution achieved enabled a near-complete protein model to be built containing 95% of the residues (table S2). The nucleotide in subunit NDUFA10 was confirmed as Mg^2+^-bound 2′-deoxyguanosine 5′-triphosphate (fig. S3B), consistent with recent biochemical data (*34*), and two modified residues were observed, the dimethylated NDUFS2-R85 (note that we use the residue numbers for mouse complex I throughout) modeled first by Agip *et al.* (*8*) and the hydroxylated NDUFS7-R87 identified by mass spectrometry (*35*). The density for NDUFS7-R87 identified it as (S)-γ-hydroxyarginine (fig. S3B), a modification that has been observed previously in structural data only as the R stereoisomer in carbon monoxide dehydrogenase from *Hydrogenophaga pseudoflava* [Protein Data Bank (PDB): 1FFV; monomer code: ARO] (*36*). In complex I, the hydroxyl points into the ubiquinone-binding channel from a conformationally mobile loop (*31*) in the central polar region of the channel (*37*). Twenty-seven phospholipids and sixteen detergents [DDM (*n*-dodecyl-β-d-maltoside)] were also modeled (fig. S3C), with one of the DDM molecules overlapping the ubiquinone-10 modeled in the C-terminal domain of NDUFA9 in the active state of porcine complex I (*12*). In the active state of bovine complex I, the same site was observed to be occupied by a phosphatidylethanolamine (*10*), suggesting it is a nonspecific site that accepts a range of hydrophobic molecules.

The densities for 2945 water molecules were identified and modeled (Fig. 1). Although most of them are located in hydrophilic, membrane-extrinsic domains, densities from well-ordered waters were observed along the central axis and E-channel and in the ubiquinone-binding channel. A total of 1763 of the waters were modeled within the 14 core subunits and 698 of those within the seven membrane-bound (ND) core subunits. For comparison, 1076 and 473 waters, respectively, were modeled in complex I from *Y. lipolytica* at 2.4 Å (PDB: 7O71) (*17*); 1587 and 615, in an open state of *O. aries* complex I at 2.3 to 2.5 Å (PDB: 6ZK9 and 6ZKA) (*13*); 1045 and 390, in a closed state of *S. scrofa* complex I at 2.4 to 2.5 Å (PDB: 7VYN and 7VYS) (*12*); 1584 and 597, in an open state of *B. taurus* complex I at 2.3 Å (PDB: 7QSM) (*10*); 1297 and 414, in an open state of *E. coli* complex I (2.3 Å; PDB: 7Z7V) (*16*); 1904 and 834, in complex I from *A. thaliana* at 2.1 Å (PDB: 8BPX) (*14*); and 972 and 392, in complex I from *C. thermophilum* at 2.8 Å (PDB: 7ZMB) (*29*). Sixty-four percent of the water locations modeled, independently, in the closed state of *S. scrofa* complex I match those found in our current model. In comparison to the predictions from simulations (*38*) on an earlier 3.0-Å resolution model for mouse complex I in the active resting state (PDB: 6ZTQ) (*39*), the overall distribution of our modeled waters matches well, except that we observe relatively fewer waters than simulated in the ubiquinone-binding channel and in a cavity in subunit ND5 that faces the intermembrane space (IMS) (Fig. 1). While the E-channel is dominated by ordered networks of charged and polar residues and water molecules, the ubiquinone-binding channel and ND5 cavity likely also contain disordered, dynamic waters whose densities are not readily observed in cryo-EM maps.

### The ubiquinone-binding channel

**Fig. 2.**
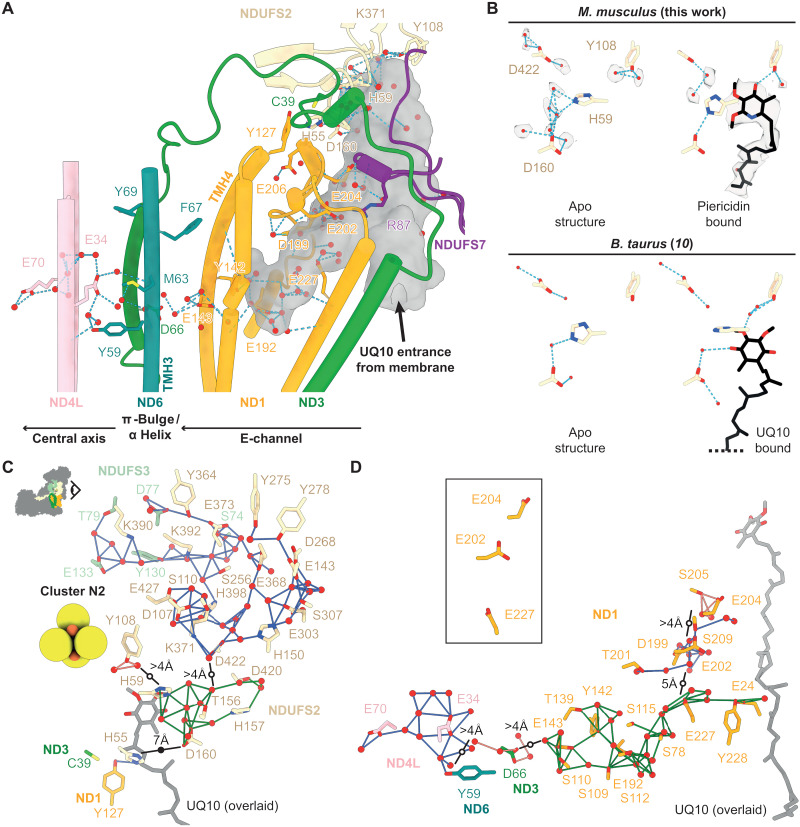
The hydrogen-bond and Grotthuss-competent networks of the ubiquinone-binding site and E-channel. (**A**) The ubiquinone-binding cavity and its leftward extension into ND1 as the E-channel, detected using a 1.4-Å probe in CASTp (*79*), are shown as a semitransparent gray surface. Water molecules (red spheres) within the cavity and around key residues are shown. Side chain and water hydrogen bonds detected by UCSF ChimeraX (*80*) are shown as light blue dashed lines. (**B**) Hydrogen-bonding networks around NDUFS2-H59, Y108, and D160 in the active state of mouse complex I and in piericidin A–bound mouse complex I (cryo-EM density shown as transparent surfaces; ChimeraX map thresholds 4.5 and 0.043, respectively) compared to the apo (UQ10-free) and UQ10-bound states of bovine complex I (PDB: 7QSL and 7QSK, respectively) (*10*). (**C**) Networks of Grotthuss-competent residues and water molecules connected to key residues (NDUFS2-H55, H59, Y108, and K371:D422) in the active site for ubiquinone reduction. Upper-left icon shows the viewpoint, with colors matching the labeled residues. (**D**) Grotthuss-competent networks in the E-channel. The view is the same as in (A). The inset shows the E202 and E204 side chains in an upward position, pointing away from E227. In (C and D), the UQ10 from PDB:7QSK (*10*) is overlaid for reference, and the links within each network are colored differently to differentiate them. Black lines indicate the gaps in the network, with distances displayed, and solid and open circles highlighting protein-obstructed and nonobstructed routes, respectively. The residues are colored according to their subunit in (A). Figures S6 and S7 present equivalent analyses to (C and D) for the closed states of complex I from *E. coli* at pH 6 (*16*) and *C. thermophilum* (*29*).

Clear densities for water molecules coordinated by cavity-lining residues are observed in the ubiquinone-binding channel, mostly in hydrogen-bonded networks at the top of the channel and in the central hydrophilic region (*37*) at the start of the E-channel (Fig. 2A). At the top, NDUFS2-H59 and NDUFS2-Y108 likely coordinate the ubiquinone headgroup and donate two protons to the nascent ubiquinol (*9*, *39*–*41*). Here, the H59 side chain is modeled in a conformation between those modeled in the UQ10-bound and “apo” active states of bovine complex I (Fig. 2B), which are related by a ~90° imidazole-ring rotation (*10*). A hydrogen-bonded water between NDUFS2-H59 and NDUFS2-D160 is similarly modeled in an intermediate position, relative to its positions in the UQ10-bound and apo models. These comparisons suggest mixed channel occupancy in our map, consistent with the ambiguous broken densities observed throughout the channel, similar to those observed in earlier active/closed structures of mammalian complex I that also lack a defined bound ligand (*8*, *10*, *13*). Here, we chose not to model these ambiguous densities as either substrate or water molecules. Two further ordered waters form hydrogen bonds to the NDUFS2-Y108 hydroxyl, as well as comparisons with the model for mouse complex I inhibited by the substrate-analog piericidin A (*39*), based on a map reprocessed here to 2.8-Å resolution (table S1 and figs. S4 and S2B), suggest that piericidin displaces one of the waters to hydrogen bond to the hydroxyl itself (Fig. 2B). Furthermore, in the presence of piericidin the NDUFS2-H59 side chain is modeled ~1.5 Å closer to NDUFS2-D160, disrupting the water network between NDUFS2-H59 and NDUFS2-D160. These subtle differences suggest that the ubiquinone headgroup displaces ordered waters from NDUFS2-H59 and NDUFS2-Y108 to form hydrogen bonds to them, ready for electron transfer from iron–sulfur cluster N2 in a (so far unobserved) reactive conformation.

Ubiquinone reduction requires two protons from the matrix, and there are several proposed uptake pathways. Here, we consider the Grotthuss-competent residues D, E, H, K, S, T, and Y (*42*) and water molecules using a distance of up to 4 Å between their protonatable N and O centers (*16*) as our criterion for evaluating candidate pathways (Fig. 2C). First, proton transfer through the highly hydrated NDUFS2 subunit was proposed by similarity to a known pathway in hydrogenases (*11*). Here, NDUFS2-Y108 is only connected to two ordered waters, but the network containing NDUFS2-H59 and D160, which extends through NDUFS2, also contains T156, H157, and T420, and 11 ordered waters and the unobstructed gap between the two networks of <5 Å suggest that thermal fluctuations or unresolved mobile water molecules may connect them. Nearby, at the channel tip, NDUFS2-K371 and D422, which are equivalent to the “canopy” residues conserved in hydrogenases at the end of their proposed proton transfer pathway (*43*), are part of an extensive network reaching toward (but not quite meeting) the solvent-accessible surface. This extensive network is also separated from the NDUFS2-H59 network by an unobstructed gap of <5 Å. Thus, in our current resting state, we do not observe any complete pathways for supplying protons from the matrix for ubiquinone reduction, but the gaps between the networks that we do observe are limited, and we note that static cryo-EM structures do not include information on the transient connections or structural and solvent rearrangements that may occur in different states. Second, a route to NDUFS2-H55, via ND3-E38 and ND1-K126, was proposed in *Y. lipolytica* complex I (*44*) and a similar route, gated by the ubiquinone-binding mode, in *Thermus thermophilus* complex I (*45*). Here, we observe NDUFS2-H55 interacting with ND1-Y127 and ND3-C39 in the “trigonal junction” characteristic of the active/closed state (*31*) and E38 interacting separately with ND1-K126 (linked by water, with 3.9-Å Glu-ɛO:LysζN distance). Again, we find no complete pathway to the matrix, and the 7-Å gap between NDUFS2-H55 and the NDUFS2-H59 network is obstructed by protein. However, during catalysis and/or deactivation, protonation changes at the trigonal junction may release C39 and alter the ND3-TMH1–TMH2 loop conformation (*31*, *46*). Last, using structures of *E. coli* complex I (*16*), the two protons for ubiquinone reduction were proposed to be taken up by different routes, one when the ubiquinone-binding site purportedly “opens” to exchange water molecules and one by ND4/ND5. The latter was proposed to traverse the central axis and E-channel to reach a triad of residues (ND1-D199, E202, and E204) and then to use ordered water molecules in the ubiquinone-binding channel to reach NDUFS2-H59. Here, ambiguous densities in the ubiquinone-binding channel between NDUFS2-H59 and ND1-D199, E202, and E204 may represent either water molecules or low-occupancy bound substrate.

### The E-channel: Connecting redox catalysis to the central axis

In our mouse structure, we observe a complete Grotthuss-competent pathway for proton transfer within the E-channel, between ND1-E24 in the ubiquinone-binding site and ND1-E143: E24–Y228–E227–a series of ordered waters–E192–S109/Y142–E143 (Fig. 2D). At the top of the E-channel, ND1-D199, E202, and E204 form a separate network, and at the bottom, ND3-D66 is also in a separate network. However, the gaps between these networks are unobstructed, and (at 4 to 5 Å) they may also be crossed by thermal fluctuations or unresolved mobile water molecules as noted above, offering a complete proton transfer route from the central axis to the ubiquinone-binding site. Notably, the modeled positions of the ND1-E202 and E204 side chains differ between our mouse structure and earlier active/closed-state structures. Although the relatively poor densities of these two carboxylate side chains (fig. S5) should not be overinterpreted, in our mouse structure and in UQ10-bound porcine complex I (PDB: 7V2C) (*12*), they are modeled pointing “upward” (away from ND1-E227; Fig. 2D, inset), whereas in our recent UQ10-bound and apo (UQ10-free; PDB: 7QSK and 7QSL) bovine structures (*10*), as well as in the closed state of *E. coli* complex I at pH 6 (fig. S6 inset) (*16*), they are modeled with their side chains flipped down toward ND1-E227. These comparisons suggest that they may switch conformation during catalysis to engage different proton transfer networks. We note that simulations on *Y. lipolytica* complex I have suggested that protons taken up to ND1-D199 from a matrix entry point close to ND1-E206 are then transferred down the E-channel for proton pumping (*17*).

An important next step for understanding complex I proton-transfer pathways and gates is to assign the protonation states of individual residues. In addition to inferring the states from hydrogen-bonding patterns or simulations, it has been proposed that only neutral carboxylates exhibit well-defined cryo-EM densities (*47*, *48*), and here, we observe clear density for both oxygens of the ND1-D199 carboxylate, plus density for a coordinated water, suggesting that it is protonated. To evaluate how generally the 2.4-Å resolution of our map reveals the protonation states of key carboxylates, we inspected the densities of 19 key Asp/Glu residues (fig. S5), noting that poor densities may also result from side chain mobility or decarboxylation, as well as deprotonation. We were unable to confidently assign any further states. Previously, densities in the closed state of ovine complex I were interpreted to suggest that ND2-E34 and ND5-E145 are protonated and ND4-E123 and ND4L-E70 are deprotonated (*13*). In our model, the ND2-E34 and ND4L-E70 carboxylates are directly hydrogen bonded to each other, and the density suggests that the proton is on ND2-E34, not on ND4L-E70, consistent with this suggestion. For the closed state of *E. coli* complex I, it was suggested (*16*) that ND1-E143, ND3-D66, and ND4L-E34 are deprotonated, and (in contrast to the ovine and mouse enzymes) ND2-E34 is also deprotonated. However, our evaluations do not allow us to draw similar conclusions and suggest caution in making such assignments. As more high-resolution data on defined biochemical states and enhanced cryo-EM analyses become available, confidence with which the protonation states of carboxylate residues can be assigned may increase.

### The central axis

**Fig. 3.**
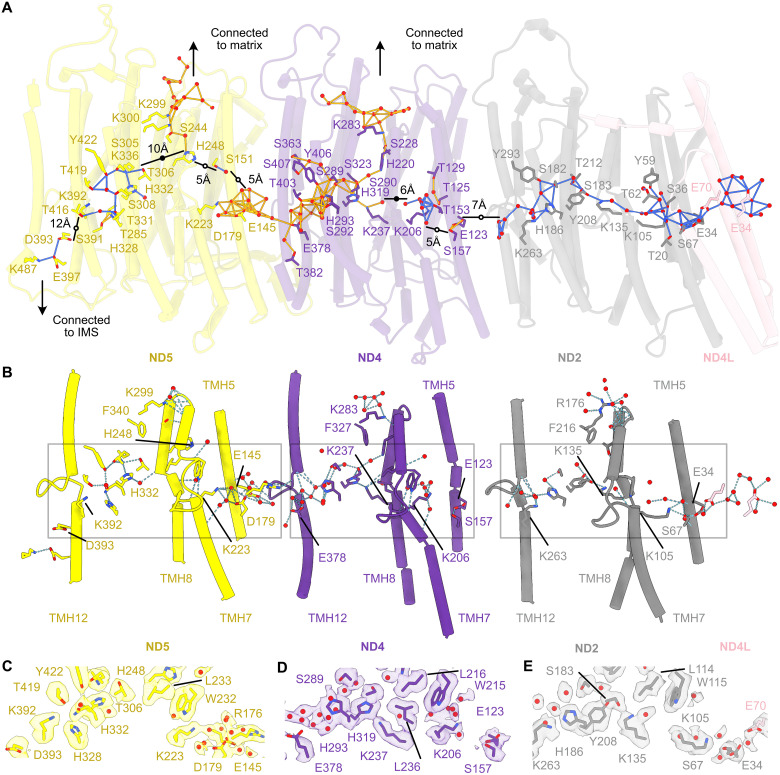
The hydrated central axis in the ND4L, ND2, ND4, and ND5 subunits. (**A**) The Grotthuss-competent networks from ND4L-E34 to ND5 across the central axis; for each network, the links are colored differently. Matrix-side connections are indicated using arrows; for simplicity, residues that extend toward the matrix beyond ND5-K299 and ND4-K283 are not shown. Black lines indicate the gaps in the network, with distances displayed and solid and open circles highlighting protein-obstructed and unobstructed routes, respectively. (**B**) Hydrogen-bonding (dashed blue lines) interaction networks between the key residues and waters of the hydrophilic axis are indicated with TMHs 5, 7 to 8, and 12 shown for clarity, and (A and B) are shown from the same viewpoint. Key residues are in stick representation, and water molecules within 4 Å of them are red spheres. (**C** to **E**) The cryo-EM (Coulomb potential) map (semitransparent surface) for the central residues in the hydrophilic axis of ND2, ND4, and ND5, respectively, as outlined by gray boxes in (B). The map depicted is in a 2-Å range of the displayed water molecules and residues, ignoring backbone atoms. The ChimeraX map threshold was set to 3.5. Figures S6 and S7 present equivalent analyses to (A) for the closed states of complex I from *E. coli* at pH 6 (*16*) and *C. thermophilum* (*29*).

In the ca. 200-Å-long central axis of conserved residues in the membrane domain, we observe water molecules distributed along the axis, filling many of the gaps between residues that are otherwise too far apart for proton transfer steps and forming Grotthuss-competent networks (Fig. 3A). Included in these networks are the TMH5-Glu and TMH7-Lys ion pairs, the central TMH8-Lys/His, and the TMH12-Glu/Lys (Fig. 3B) residues that are known to be crucial for catalysis (*21*–*28*).

The networks and connectivities across the three antiporter-like subunits vary considerably. In the antiporter-like subunit interfaces, ND2-TMH5-E34 is connected to ND4L-E70 and ND5-TMH5-E145 to ND4-TMH12-E378, but ND4-TMH5-E123 is separated from ND2-TMH12-K263 by 7 Å (Fig. 3A); as the gap is unobstructed, mobile water molecules may be present, consistent with weak cryo-EM densities observed there. In all three subunits, the TMH5-Glu-ɛO and TMH7-LysζN pairs are too far apart (5 to 6 Å) for tight ion-pair interactions, although connections are established in ND2 and ND5 by intervening residues and waters. The ND2-E34–K105 gap is bridged by a hydrogen-bond network including S67 and one water (Fig. 3, A, B, and E). In ND4, S157 (equivalent to ND2-S67) sits between E123 and K206, but K206 is surrounded by waters and pointing away (Fig. 3, A, B, and D), and it also lacks onward connections to TMH8-K237 as the 6-Å onward-gap in the network is obstructed. In ND5, D179 (in a similar location to ND2-S67 and ND4-S157) forms a direct hydrogen bond to E145, and both E145 and D179 are linked to K223 and R179 by hydrogen-bonding networks involving intervening water molecules (Fig. 3, A to C). Thus, all three canonical ion pairs are stabilized by hydrogen-bonding networks and observed in intermediate states between the closed and open ion-pair conformations simulated in *T. thermophilus* ND4 (TMH5-Glu-ɛO to TMH7-LysζN ~4 and ~9 Å, respectively) that depended on the TMH8-Lys protonation state (*38*, *49*, *50*).

In the center of ND2, TMH8-K135, TMH10-S183, and a nearby water (Fig. 3, A, B, and E) extend the Grotthuss-competent ND2 network across the entire subunit (Fig. 3A), whereas the hydrophobic ND4-W215, TMH7b-L216, and TMH8-L236 residues interrupt the ND4 network (Fig. 3, A, B, and D) between TMH7-K206 and TMH8-K237. Because of an insertion in ND4 (relative to ND2), L236, not TMH8-K237, occupies the position of ND2-TMH8-K135; instead, K237 interacts with ND4-TMH11-H319, water, and the exposed A233 carbonyl in the TMH8 π-bulge, and H319 interacts with TMH10-S290 (Fig. 3, A, B, and D). The implications of the residue insertion breaking the ND4 network are unclear: In *T. thermophilus*, there is no insertion and the configuration of ND4-TMH8 matches that observed here in ND2; in *E. coli* ND4, the TMH8-Lys side chain and preceding main chain conformation were observed to vary between structures (*16*). After the break, our mouse ND4-TMH8-K237 residue begins a continuous “onward” Grotthuss-competent network to ND4-TMH12-E378 (and ND5-E145; Fig. 3A). ND4-H319 is further connected to the matrix via H220, thus connecting this whole residue network to the matrix (a connection that is absent from ND2). In ND5, there is a 5-Å unobstructed gap in connectivity that may complete the onward connection from ND4 and ND5-E145 to ND5-H248, which is also connected to the matrix, via S244 and K299. In ND5, there is no TMH8-Lys, and the equivalent space to that occupied in ND4 by TMH8-K237-ζN is occupied by water, stabilized by TMH11-H332 and the ND5-TMH8 π-bulge (Fig. 3, A to C) (*11*). ND5-H248 is on the π-bulge, and it has been suggested to switch between two positions, through rearrangement of the π-bulge, both in complex I and in the related Mrp-type antiporters (*16*, *51*, *52*), to direct protons from the matrix either toward TMH7-K223 or to ND5-TMH12-K392. Here, it is configured for proton transfer toward K223, with a 10-Å gap between the H248 and K392 networks that is obstructed by intervening protein (Fig. 3A). The axis terminates in ND5 at a pair of charged residues, TMH12-K392 and D393 (that are isolated from each other because of their rotamer positions). Although water molecules hydrating these residues were not observed, perhaps because of poorer local resolution in this region (fig. S2), the apparent 12-Å gap between the networks is unobstructed and D393 is connected to E397 and K487, which appears accessible to bulk solvent in the ND5 IMS-facing cavity.

### Proton-pumping uptake and exit pathways in the antiporter-like subunits

**Fig. 4.**
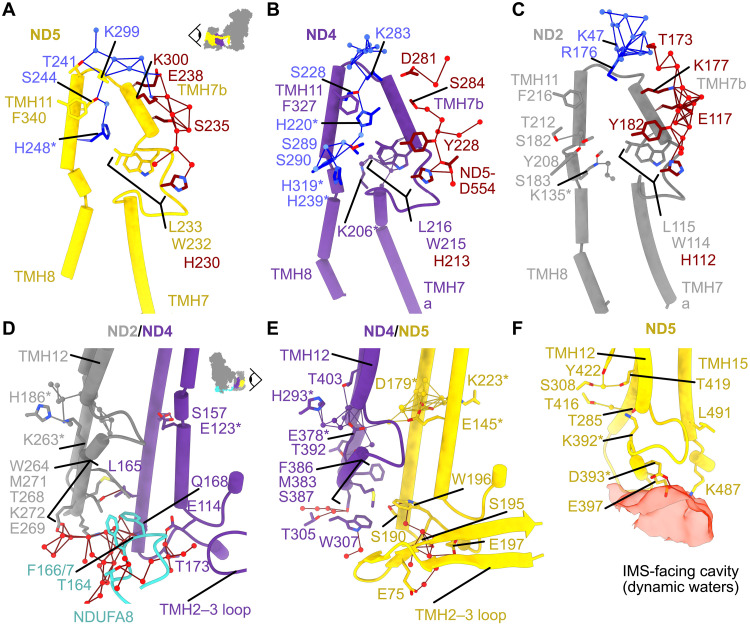
Investigating proton uptake and exit pathways in the antiporter-like subunits ND2, ND4, and ND5. (**A** to **C**) TMHs 7 to 8 for ND5 (A), ND4 (B), and ND2 (C) are viewed along the membrane domain from the toe of the complex (see inset viewpoint). The residues (sticks) and waters (spheres) of the Grotthuss-competent networks that are connected to the matrix between TMH10 and TMH11 are shown in blue, and those that are part of the network on the outside of TMH7b are shown in red. Key residues that are part of the central axis are indicated with asterisks. (**D** to **F**) The subunit interfaces (viewed from the opposite side of the membrane domain to the transverse helix; see inset viewpoint) between ND2-TMH12 and ND4-TMH5, 6 (D); ND4-TMH12 and ND5-TMH5, 6 (E); and ND5-TMH and ND5-TMH15, where TMH15 substitutes for the “next subunit” (F). The IMS-facing cavity of ND5-containing dynamic waters is indicated by a red semitransparent cavity detected using a solvent probe (*79*), with the surface 7 Å from the displayed residues. Residues and waters of the Grotthuss networks at the subunit interfaces are shown as sticks and spheres, respectively, with waters in networks located in the IMS in red. ND2-K263, ND4-E378, and ND5-K392 are the TMH12 proton release sites in the central axis.

In our mouse structure, Grotthuss-competent networks connect the central axis in ND4/ND5 to the matrix [Figs. 3A and 4, A to C (blue)]. Both connections are to a spatially conserved His (ND4-TMH7b-H220 and ND5-TMH8-H248), adjacent to a fully conserved TMH11-Phe (ND4-F327 and ND5-F340) and underneath a fully conserved TMH10-Lys (ND4-K283 and ND5-K299) (*16*, *17*). No matching connection is observed in ND2, where ND2-TMH7b-T119 (a Tyr in many species) and ND2-TMH8-L132 are found in the sequence positions of the ND4/5-His. The TMH11-Phe is conserved as ND2-TMH11-F216, but ND2-TMH10-R176 is found in place of the ND4/5-TMH10-Lys, and the adjacent ND2-K177 residue does not provide a substitute because it points up and away from F216 (Fig. 4, A to C). We note that the TMH11-Phe residues have been suggested to control hydration because simulations on *Y. lipolytica* complex I in which ND4-TMH11 (that harbors *Yl*F343) was modeled according to ND2-TMH11 (that harbors *Yl*F324) resulted in increased ND4 hydration (*17*). However, the ND2 and ND4 TMH11-Phe residues match closely between the mouse and *Y. lipolytica* structures, and in mouse, we observe the opposite subunit hydration pattern: a connection to the matrix in ND4 (not ND2) and less hydration in the ND2 subunit core. Another suggestion is that ND2 does not take protons up from the matrix at all (*13*, *16*). It is also possible that the enzyme rests with its ND4/5 matrix channels open and ND2 channel closed, or the ND2 channel may form only transiently. Simulations have also proposed hydration of TMH7b in all three subunits (*38*), and we observe numerous waters on the external face of the TMH7b helices as they descend to the ND5 transverse helix, above the intra-TMH7 loops [Fig. 4, A to C (red)]. These waters lie outside the subunit cores, above the membrane interface, but exhibit well-ordered densities, and they may mark proton-entry points. In each subunit, a conserved Leu residue [ND2-L115, ND4-L216, and ND5-L233, within the Leu-Trp-His “gates” marked in Fig. 4 (A to C)] lies en route to the central axis from the putative entry points, adjacent to the intra-TMH7 loops. The Leu conformations were observed to correlate with TMH7b-hydration in *Y. lipolytica* complex I, where the ND2 gate was observed fully open alongside increased internal hydration (*11*, *17*). The Leu residues have also been implicated in proton gating in simulations (*38*, *50*, *53*), and the Leu-to-Ala variant in *Paracoccus denitrificans* ND4 (*28*) was inactive. Here, all three Leu gates are closed.

The IMS-facing ND5 cavity, which extends toward the central axis but makes no connection to it (Fig. 4F), is expected to be water-filled and the proton-release site from TMH12b-K392 (*51*, *53*–*55*). All four pumped protons have been proposed to exit here (*13*, *16*, *17*), or, supported by simulations, transient channel opening to the IMS may occur along TMH12b in ND2 and ND4 also (*38*, *49*). Considering the relationship between TMHs 4 to 8 and 9 to 13 (*56*), TMH12b is the symmetry partner of well-hydrated TMH7b. However, although ND2-TMH12-K263 and ND4-TMH12-E378 are hydrated in our structure (Fig. 3), there are no ordered water molecules/protonatable residues observed tracing out exit pathways, and hydrophobic residues (on ND2/4-TMH12, or TMH5, 6 and the TMH6–TMH7 loop of ND4/ND5) partition them from IMS-hydrated regions (Fig. 4, D and E). In addition, the ND5-TMH2–TMH3 hairpin loop is tightly associated with the bottom of the ND4/ND5 interface; whereas the shorter ND4-TMH2–TMH3 hairpin loop does not reach the ND2/ND4 interface in our mouse structure, the C terminus of supernumerary subunit NDUFA8 substitutes for it. In *E. coli* and *T. thermophilus* complex I, the ND4-TMH2–TMH3 hairpin loops are longer, like the ND5 loop (*9*, *15*, *56*), and only the ND2-TMH2–TMH3 loops (which extend toward ND4L/ND6) are shorter. The hairpin loops and/or residues of the TMH12b of ND5/ND4 at these interfaces may help regulate proton exit from ND4/ND2-TMH12b. Last, water molecules have recently been observed between the central axis and the IMS in ND2 of *A. thaliana* complex I (*14*), suggesting an exit pathway between TMHs 10, 11, and 12b. Here, we observe water (N552 in PDB: 8OM1) coordinated between ND2-H186 and ND2-TMH12-K263 (*A. thaliana* H326 and K310), two conserved residues in the central axis (Fig. 3A), at the top of the proposed pathway. However, only a single water density is observed in the pathway region as it descends to the IMS (N557 in PDB: 8OM1, adjacent to ND2-I266); a single water molecule was modeled in the same position in complex I from *S. scrofa* (PDB: 7VYS) (*12*). The lower number of waters observed in mouse relative to that in *A. thaliana* is consistent with the lower proportion of hydrophilic residues surrounding the proposed pathway in the mouse enzyme and their low sequence conservation between species.

### Uptake and exit pathways for the “fourth” pumped proton

**Fig. 5.**
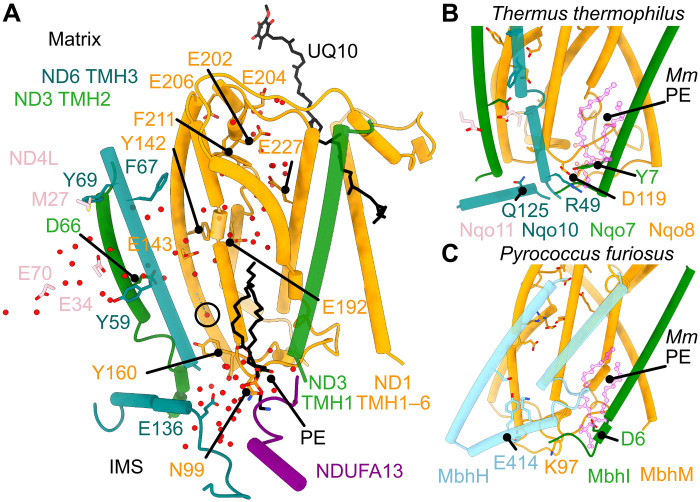
The hydrated E-channel connecting the ubiquinone-binding cavity to the central axis and a potential proton exit route to the IMS. (**A**) Charged residues that form the E-channel in ND1 (E206, E204, E202, E227, E192, and E143, orange) lead from the ubiquinone-binding site into the center of the membrane. E143 interfaces with ND3-TMH2 and ND6-TMH3, and ND3-D66 (green) bridges the connectivity of water molecules between ND1 and ND4L, around the α-helical ND6-TMH3. From here, ND4L-E34 and E70 lead into ND2 (not shown). Water molecules within 5 Å of the polar residues indicated are shown as spheres. A tentative proton-exit route to the IMS is highlighted by residues ND1-Y160 and N99, ND6-E136, and NDUFA13, with Y160 as a potential gating residue for the network, reaching up toward the water molecule circled (H615 in PDB: 8OM1). The bound phosphatidylethanolamine (PE) lipid, supported by NDUFA13, may gate proton transfer and/or its phosphate group may be involved. The ubiquinone-10 (UQ10) used as a reference is from the bovine enzyme in the active state (PDB: 7QSK) (*10*). (**B**) Comparison with *T. thermophilus* complex I (*59*). The labeled residues were implicated in a proton-exit route to the cytoplasm/IMS predicted in a simulation (*42*), with the N-terminal extension of Nqo7/ND3 contributing Y7, in the same position as the PE phosphate group in A (overlaid in magenta). (**C**) Comparison with *Pyrococcus furiosus* membrane-bound hydrogenase (*61*). Equivalent residues have been matched where possible in MbhM (ND1) and MbhI (ND3). ND5-like MbhH TMH12 and TMH13 are shown, with conserved axis residues and the broken TMH12 motif. As in (B), D6 on an N-terminal extension of MbhI (ND3) occupies the same position as the lipid phosphate in mouse (overlaid in magenta), which is suggested to hydrate in simulations and form a possible proton pathway to the cytoplasm/IMS (*81*).

If the membrane domain comprises four independent proton-pumping modules, then the elusive fourth module is likely contained within ND1/ND3/ND6/ND4L (*9*, *13*, *17*, *18*, *38*, *42*, *53*, *56*, *57*), where protons taken up from the matrix may be transferred to the central axis through the E-channel. Proposed exit pathways include the ND1/ND3 and ND6/ND4L/ND2 interfaces, although they contain hydrophobic residues, are poorly conserved, and lack well-defined densities for ordered water molecules. Here, we have modeled a single water (H615 in PDB: 8OM1) at the ND1/ND3/ND6 interface (between ND1-TMH3, ND1-TMH4, ND3-TMH2, and ND6-TMH3), weakly stabilized (part-way between ND1-E143 and ND1-TMH3-Y160) by a hydrogen bond to the backbone carbonyl of ND1-A147 (Fig. 5A). Y160, which is replaced by Phe in the *B. taurus* and *O. aries* enzymes but retained in that from *S. scrofa*, does not contact the water but creates a hydrophobic barrier to the IMS. A matching water molecule was also modeled in complex I from *S. scrofa* (PDB: 7VYS) (*12*), suggesting that the Tyr may help create a more favorable environment. The interface is stabilized by a conserved phospholipid (*8*, *10*, *11*, *13*, *58*), adjacent to ND1-TMH3, and encased by ND3-TMH1 and supernumerary subunits NDUFA13 and NDUFA1. The lipid may prevent proton leak and facilitate conformational changes. In *T. thermophilus* complex I (*59*) and cyanobacterial NDH/NDH-1L (*60*) the lipid is replaced by an N-terminal extension to ND3-TMH1 that contains a conserved Tyr overlaying the position of the phosphate (Fig. 5B). Furthermore, in the complex I-homologous membrane-bound hydrogenases (*61*), where the antiporter-like domain is rotated 180° to form an ND5/ND1 interface (Fig. 5C), the equivalent ND5-TMH12 proton-exit site (Fig. 4F) overlays the phospholipid, along with an N-terminal Asp-containing extension to ND3-TMH1. This region of the complex deserves further attention for its potential role in catalysis, perhaps as a potential proton exit route to the IMS.

## DISCUSSION

Pathways for supplying the chemical protons for ubiquinone reduction have been proposed through NDUFS2 (*11*), at the ND3-TMH1–TMH2 loop (*44*, *45*), and from ND4/5 along the central axis, E-channel, and ubiquinone-binding channel (*13*, *16*). Here, we explored structurally defined Grotthuss-competent networks at the top of the ubiquinone-binding channel but could not identify any complete pathway to the matrix in our active resting state. Pathways may form transiently or in specific catalytic intermediates, and short gaps in the defined networks may, when not obstructed by protein, be closed by mobile water molecules. However, we did observe extensive networks of protonatable residues and waters to residues at the top of the channel that constitute a substantial reservoir of proton donors (Fig. 2C); similar networks are observed in the structures of the closed states of complex I from *E. coli* at pH 6 (*16*) and “state 2” of complex I from *C. thermophilum* (*29*), in which water molecules were also modeled (figs. S6A and S7A). We must therefore consider not only the potential routes by which proton uptake could occur but also whether a specific route is used, and if so, how protons are taken up only along this route, not just from the nearest available source. For example, how would the reprotonation of a high-energy “proton hole,” generated on NDUFS2-H59/Y108 upon ubiquinol formation, be enforced to occur long distance from ND4/5, rather than from local networks? One interesting idea is for local reservoirs to become depleted by supplying the protons for “priming” redox turnovers that do not themselves drive proton pumping, but either empty off-pathway proton reservoirs or create energized pathway states that promote proton transfer steps and induce network connections. This idea may explain the delayed onset for proton pumping relative to redox catalysis observed in *E. coli* complex I (*62*), but it is not consistent with the proposal that the ubiquinone-binding site opens to exchange water molecules and reequilibrate with the matrix during every cycle of catalysis (*13*, *16*). We note that simulations have suggested how UQ10 binding is accommodated by migration of 30 to 40 water molecules out of the ubiquinone-binding channel; the waters were predicted to diffuse through the protein structure into the matrix on a tens-of-nanosecond time scale, suggesting that this process does not limit the rate of turnover (*63*).

Defining how protons are taken up for ubiquinone reduction is central to the mechanism of energy transduction. In the mechanism of Kravchuk *et al.* (*16*) proton uptake is intrinsic to energy conservation, with the redox free energy delivered to the central axis as a single, negatively charged, high-energy proton hole, which is subsequently filled by proton uptake by ND4/5. However, in neither our current structure (Fig. 2D) or the closed states of complex I from *E. coli* at pH 6 (*16*) and *C. thermophilum* (figs. S6B and S7B) (*29*) is there a complete network configured for this proton transfer “delivery” event. In our mouse structure, the main E-channel network connects ND1-E143 to ND1-E24, in the middle of the ubiquinone-binding channel. Similarly, in the *E. coli* structure, it progresses from ND3-D66 but diverges from the mouse pathway in NuoH/ND1 because *Ec*V206 replaces *Mm*E192 and *Ec*H208 replaces *Mm*N194. In the *E. coli* structure, the Grotthuss-competent network extends to *Ec*D223 (*Mm*S209), at a different position in the central section of the binding channel (fig. S6B). Separate *E. coli* networks connect E227 to E24 and E202 and D199 and E204 (via a series of waters) to NDUFS2-H59. In the *C. thermophilum* structure, the Grotthuss-competent network extends from ND2-K105 in the central axis to ND1-E24 (the same residue as in mouse) but no further (fig. S7B). Therefore, in none of these structures is there a complete, structurally defined pathway for proton transfer between the ubiquinone-binding His/Tyr ligands and ND3-D66.

Delivering all the redox energy to the membrane domain as a single extremely proton-hungry proton hole (energetically equivalent to Δp*K*_a_ = 12) also raises the question of how the reactive intermediates are protected from quenching by local proton transfer events. Stuchebrukhov and Hayashi (*64*) also suggested that all the redox energy is transferred to the membrane domain by a single proton traversing the E-channel, but instead of using water molecules to transfer the proton up the ubiquinone-binding channel, they proposed the anionic quinol (QH^−^) intermediate steps part way down, to the top of the E-channel, perhaps ready for proton uptake from the conformationally mobile ND1-E202/E204 residues (insets in Fig. 2D and figs. S6B and S7B) or from ND1-E24, connected to the main E-channel network in our mouse structure (Fig. 2D). Alternatively, proton uptake may be a simple process, not itself directly harnessed for proton pumping. Proton uptake through NDUFS2 is more likely in this category but would require a separate mechanistic feature to capture the redox energy. Kaila (*55*) proposed that the bound ubiquinone is reduced isopotentially with NADH oxidation, storing the energy for proton pumping in tight ubiquinol binding (*K*_D_^QH2^ << *K*_D_^Q^) until ubiquinol release. Notably, in Kalia’s mechanism, a single proton travels down the E-channel (i.e., in the opposite direction to the two mechanisms above) (*16*, *64*) to initiate proton pumping, and here, we suggest a tentative output channel for this proton (Fig. 5). Parey *et al.* (*17*) further suggested two protons travel down the E-channel, injected by a sequence of ubiquinone binding and redox reactions. Defining the direction of proton transfer through the E-channel is thus a key unanswered question for complex I catalysis.

Once the redox energy has been transferred to the central axis, there are two opposing models for proton pumping: Four separate modules connected in series each pump one proton, or the whole membrane domain acts as a single four-proton transferring unit. Two structural properties may distinguish them: (i) In four-module schemes, each module has its own proton uptake and exit channel, whereas single-unit schemes need only one uptake and one exit channel. (ii) In four-module schemes, protons do not transfer between modules, whereas in single-unit schemes, they do. Kaila (*55*) proposed a four-module model in which two “waves” propagate along the membrane domain. Taking ND2 as an example, the forward wave opens the Glu–Lys ion pair, displacing a proton from TMH8-Lys to TMH12-Lys, and closing the TMH8-Lys-matrix uptake channel, and the backward wave releases the proton to the IMS, recloses the ion pair, and reopens the matrix channel. Alternatively, Kravchuk *et al.* (*16*) proposed that five protons are taken up from the matrix to the central axis through ND4/ND5. One fills the proton hole generated by ubiquinone reduction, and four are released through ND5; protons transfer along the length of the membrane domain, with ND2 acting only as a proton conduit and temporary proton-storage device. Although this model is challenged by data from a variant of *Y. lipolytica* complex I that lacked subunits ND4 and ND5 but was reported to still pump two protons per NADH oxidized (*65*), no further studies have been performed to further pursue its mode of catalysis, and biochemical and biophysical strategies to distinguish the two opposing models being discussed are currently lacking.

1) In our structure, we observe complete Grotthuss-competent proton transfer connections to the matrix from the central axis in ND4 and ND5 (Fig. 3A), although similar connections are not present in either of the closed states of complex I from *E. coli* at pH 6 (*16*) or *C. thermophilum* (figs. S6C and S7C) (*29*). No matrix connection is observed in ND2, and it has been suggested that no connection can form because ND2 lacks the His–Polar residue–Lys motif found in ND4/ND5 (*16*). However, note that *Ec*ND2 does contain the “Polar” S239 residue (equivalent to the *Ec*ND4-D258 and *Ec*ND5-S250 motif residues) and the limitation is the larger Y231 in the “His” position. A Tyr–Polar–Lys motif is also present in *Y. lipolytica* ND2, which shows extensive hydration but lacks a complete Grotthuss network to the matrix due to the rotamer conformation of its "Polar" *Yl*T233 (*17*). Therefore, ND2 matrix connections may be transient. The uptake pathway in the fourth putative module is also unclear; one possibility is uptake to ND1-D199 and then transfer down the E-channel (*17*). For the exit channels, only the substantial water-filled cavity in ND5 is clearly defined in current structures. However, simulations have suggested that hydrated channels form transiently to transfer protons from the ND2/ND4-TMH12-Lys/Glu residues to the IMS. Waters suggestive of such a channel have recently been reported in *A. thaliana* complex I (*14*), and, here, we have identified a region of interest for the fourth putative exit channel (Fig. 5). Thus, regarding proton uptake and exit channels, the preference for a four-module or single-unit model rests on whether all the channels need be clearly visible in structural data. We note that proton exit channels in cytochrome *c* oxidase that must exist are not clearly visible (*66*), presumably being constituted only transiently or in different functional states.

2) What is the structural evidence for proton transfer along the length of the membrane domain, as proposed in single-unit models (*13*, *16*)? In our mouse resting-state structure, there is a continuous Grotthuss-competent network from ND4L across ND2 and from the ND4-TMH8-Lys to the ion pair in ND5, but otherwise, the connectivity is fragmented, particularly with two protein-obstructed gaps in ND4 and ND5 (Fig. 3A). Like the matrix connections, the trans-subunit connections suggest a difference between ND2 and ND4/5 that may reflect either their fundamental properties or the state that the enzyme is resting in. We note that the closed state of *E. coli* complex I at pH 6 (*16*) displays no defined proton transfer connections between the antiporter-like subunits (fig. S6), and the closed state of complex I from *C. thermophilum* (*29*) displays networks that are also fragmented but that includes intersubunit connections from ND4L to ND2 and from ND2 to ND4. Thus, regarding long-distance, longitudinal proton transfer events, the preference for a four-module or single-unit model rests on the emphasis and interpretations placed on the partial connections observed in current structures.

In summary, fundamental questions about the mechanism of energy transduction by complex I remain unanswered. Is proton uptake for ubiquinone reduction the driving step for proton pumping? What is the role of the E-channel: Does it transport protons from the ubiquinone-binding channel to the central axis or protons from the central axis for ubiquinone reduction? Do protons transfer freely along the central axis, with the membrane domain acting as a single unit, or does the domain consist of four discrete transport modules? In the latter case, how is energy transferred between them? How are proton uptake and exit controlled and gated, enforcing directional transfer against Δ*p* and avoiding side reactions that quench reactive intermediates and compromise energy conservation? Detailed structural data on defined states on the catalytic cycle combined with biochemical, biophysical, and computational investigations are required to answer these questions.

## MATERIALS AND METHODS

### Purification of mouse complex I

All experimental procedures were carried out at 4°C, unless otherwise stated. For the sample imaged, C57BL/6 mice (36 in total) were euthanized by cervical dislocation following the U.K. Animals (Scientific Procedures) Act, 1986 (PPL: 70/7538, approved by the local ethics committees of the MRC Laboratory of Molecular Biology and the University of Cambridge and by the U.K. Home Office). Hearts (ca. 0.20 g each) were surgically removed and placed in cold AT buffer containing 10 mM tris-HCl (pH 7.4), 75 mM sucrose, 225 mM sorbitol, 1 mM EGTA, 0.1% (w/v) fatty acid–free bovine serum albumin (Merck Millipore), and 1× (one tablet per 50 ml of buffer) cOmplete EDTA-free, protease-inhibitor tablet (Roche). Then, mitochondria were prepared using methods adapted from those described previously (*8*). Hearts were cut into small pieces and washed and then homogenized (1000 rpm for 10 strokes with a Teflon pestle in a 30-ml Wheaton glass homogenizer with 10 ml of AT buffer per gram of tissue). The homogenate was centrifuged (1000*g* for 5 min) to remove cellular debris; then, crude mitochondria were collected by a further centrifugation (9000*g* for 10 min). The pellet (ca. 44-mg protein) was resuspended in buffer containing 20 mM tris-HCl (pH 7.4), 1 mM EDTA, 10% glycerol, and 1× cOmplete EDTA-free protease-inhibitor tablet and stored at −80°C. Mitochondrial membranes were isolated as described previously (*8*). Briefly, thawed mitochondria were diluted in resuspension buffer to 5 mg ml^−1^ then ruptured on ice by sonication (three 5-s bursts each followed by 30 s rest; 65% amplitude setting; Q700 Qsonica Sonicator). The membranes were collected by centrifugation (75,000*g* for 1 hour), yielding ca. 24-mg protein that was resuspended in the same buffer at 5-mg protein ml^−1^ and stored at −80°C.

Complex I was prepared as described previously (*8*). Membranes were solubilized with DDM at a detergent:protein ratio (w/w) of 2.2:1 [final 1% (w/v) DDM from a 10% stock] for 30 min with continuous stirring on ice. Nonsolubilized material was removed by centrifugation (48,000*g* for 30 min); then, the supernatant was applied to a Hi-Trap Q HP anion exchange column (Cytiva), pre-equilibrated in buffer A [20 mM tris-HCl (pH 7.14) at 23°C, 1 mM EDTA, 0.1% DDM, 10% ethylene glycol (v/v, VWR), 0.005% asolectin (Avanti), and 0.005% CHAPS (Calbiochem)] at 0.3 ml min^−1^. The column was washed with 1.5 column volumes of buffer A then 3 column volumes of 20% buffer B (buffer A + 1 M NaCl); then, complex I was eluted at 35% buffer B. The complex I–containing fractions (1.4 ml) were pooled, concentrated to ca. 100 μl [100-kDa MWCO (molecular weight cut-off) Amicon Ultra Centrifugal filter, Merck Millipore], and was applied to a Superose 6 Increase 5/150 size-exclusion column (Cytiva) pre-equilibrated in buffer C [20 mM tris-HCl (pH 7.14) at 23°C, 150 mM NaCl, and 0.05% DDM] at 0.03 ml min^−1^, and 30-μl fractions were collected. Chromatography was performed using ÄKTA micro FPLC systems (Cytiva) and monitored at 280 and 420 nm. Protein concentrations were estimated at 280 nm (ɛ = 0.2 mg ml^−1^ cm^−1^). The total yield was 0.5 mg of complex I, with a peak concentration of 4 mg ml^−1^. An NADH:decylubiquinone oxidoreductase activity of 19.4 ± 0.4 μmol min^−1^ mg^−1^ (SD, *n* = 4) was determined on a matching sample in a 96-well SpectraMax 384 plate reader at 32°C: Complex I (0.5 μg ml^−1^) was incubated with 100 μM DQ in 0.075% (w/v) asolectin and 0.075% (w/v) CHAPS in 20 mM tris-HCl (pH 7.5 at 20°C); the reaction was initiated by 100 μM NADH and monitored at 340 to 380 nm (ɛ = 4.81 mM^−1^ cm^−1^).

### Cryo-EM grid preparation, screening, and imaging

Cryo-EM grids were prepared as described previously (*8*, *30*). UltrAuFoil gold grids (0.6/1; Quantifoil) (*33*) were glow-discharged at 20 mA for 90 s under vacuum, incubated for 48 hours under anaerobic conditions at room temperature in 5 mM 11-mercaptoundecyl hexaethylene glycol (SPT-0011P6, SensoPath Technologies) (*32*), then washed in ethanol, and air-dried. Complex I (3 μl per grid, ca. 4 mg ml^−1^) was applied to each grid in an FEI Vitrobot Mark IV (Thermo Fisher Scientific) at 4°C and 100% relative humidity. The grids were blotted for 10 s under a force setting of −10 and frozen by plunging into liquid ethane.

Grids were screened at the Department of Biochemistry, University of Cambridge using a Titan Krios cryo–electron microscope (Thermo Fischer Scientific); then, high-resolution images were recorded using a Titan Krios microscope at the U.K. national electron Bio-Imaging Centre (eBIC), at the Diamond Light Source. The instrument was operated at 300 kV using a Gatan K3 detector in superresolution electron counting mode at a nominal sampling rate of 0.5425 Å pixel^−1^. Non–gain-normalized images were acquired with a post-column imaging energy filter (Gatan BioContinuum) set to a slit width of 20 eV at a dose rate of ca. 15.8 electrons Å^−2^ s^−1^. The sample was exposed to a total dose of ca. 40 electrons Å^−2^ in a 2.53-s exposure over 40 individual movie frames. The 70- and 100-μm C2 and objective lens apertures, respectively, were used during imaging. SerialEM was used to operate the microscope and to automate the data collection (*67*). Images were collected with a targeted defocus range of −0.8 to −2.0 μm, with an autofocus routine at every acquisition area.

### Cryo-EM data processing for the active state of mouse complex I

Cryo-EM data processing was carried out in RELION-3.1 (*68*) unless otherwise stated (fig. S1). Superresolution movies were gain-corrected and down-sampled to 1.085 Å pixel^−1^. Beam-induced motion and stage drift in the gain-normalized movie frames were corrected using RELION’s implementation (*69*) of MotionCor2 (*70*) and per-micrograph contrast transfer function (CTF) parameters determined from the non–dose-weighted motion-corrected movie sums with CTFFIND-4.1 (*71*). Automated selection of particles was carried out using crYOLO v.1.3.5 (*72*), in combination with a model trained on 12,192 manually curated coordinates from 100 micrographs. The process yielded 357,798 coordinates from a total of 2674 micrographs. The micrographs were then manually inspected, and 306 were discarded because of nonvitreous ice, contamination, or poorly modeled CTFs, reducing the number of coordinates to 307,866 from 2368 micrographs. Then, in RELION-3.1, particles were extracted from the curated micrographs at a sampling rate of 4.9 Å pixel^−1^ in 100-pixel boxes (4.5× down-sampled), and the down-sampled particle images were refined against a previously determined reconstruction of the active state of mouse complex I (EMD-4345), scaled to 4.9 Å pixel^−1^ (*8*). The particles were then three-dimensionally (3D) classified into 15 classes, without particle alignment, using the consensus orientations determined by the refinement and a regularization parameter (*T*) of 20. Particles in classes not resembling complex I, or with resolution estimates <10 Å, were discarded, leaving 270,698 particles. The remaining particles were reextracted at 2.1 Å pixel^−1^ in 230-pixel boxes (1.9× down-sampled), refined and 3D-classified as above into 15 classes; a further 49,256 particles in classes with low signal-to-noise ratios were removed. The remaining 221,442 curated particles were reextracted at the nominal sampling rate of 1.085 Å pixel^−1^ in 450-pixel boxes and refined using a soft-edged mask encompassing protein regions only. Last, they were resolved into homogeneous subsets by 3D classification without particle realignment into eight classes, and the best-resolved classes [that were all found to represent the active state by comparison to a previously reported structure (*8*)], representing 109,866 particles, were refined, resulting in a reconstruction at an estimated resolution of 3.5 Å. Focused 3D classification attempts around the ubiquinone-binding site were unsuccessful. The particles were reextracted and recentered, according to their refined translational offsets, and refined again. Then, they were subjected to iterative cycles of CTF parameter refinement to estimate the per-particle defocus and refined to yield a reconstruction with an estimated resolution of 2.8 Å. An additional three cycles of CTF parameter refinement, including corrections for the effects of per-particle astigmatism and beam-tilt, were performed, improving the resolution to 2.7 Å. Then, the particles were subjected to Bayesian polishing (*69*), leading to reconstructions with estimated resolutions of 2.6 Å. The polished particles were then subjected to further rounds of CTF parameter refinement, including corrections of anisotropic magnification and higher-order microscope aberrations (*68*), to generate a reconstruction with an estimated resolution of 2.5 Å. The calibrated sampling rate was determined from this reconstruction, by comparison to a previously determined model of mouse complex I (*39*), to be 1.068 Å pixel^−1^. Bayesian polishing was repeated (using the previously determined particle trajectories and per-frame resolution dependent weightings) to rescale the particles to a finer sampling rate of 0.6975 Å pixel^−1^ in 700-pixel boxes (1.3× bin; calibrated pixel size of 0.6866 Å pixel^−1^). CTF parameter refinement, including per-particle defocus estimation, corrections for per-particle astigmatism, and higher-order aberrations, was repeated, and the particles were refined to yield a reconstruction with an estimated resolution of 2.4 Å.

Global resolution was estimated from the Fourier shell correlation (FSC) between two independent, unfiltered half-maps, according to the FSC 0.143 criterion (*73*). The unfiltered half-maps were postprocessed in RELION with an additional argument *--*autob_highres 2.176 (2.176 Å corresponds to the highest local resolution determined from RELION LocalRes) to filter them according to the estimated resolution and to automatically estimate and apply a sharpening B-factor (*73*). The model-generated mask used for resolution estimation was generated in UCSF Chimera v.1.13.1 (*74*) using the molmap function, before being low-pass–filtered to 20 Å, extended by 3 pixels, and having a 5-pixel soft cosine edge added using RELION MaskCreate. Local resolution was estimated by iterating the RELION’s LocalRes tool. The consensus map was locally sharpened from the unsharpened, unfiltered half-maps generated from RELION postprocess (user-provided B-factor and ad hoc low-pass filter set to 0 and Nyquist, respectively) using phenix.autosharpen (Phenix v.1.18.2-3874) (*75*), setting the resolution limit to the highest local resolution determined from RELION LocalRes (2.176 Å), and with a local sharpening box size of 15 × 15 × 15 pixels and a targeted overlap of 5 pixels. This map was used for model building and refinement.

### Cryo-EM data reprocessing for piericidin-bound mouse complex I

For the piericidin-bound map, two datasets from the same preparation but recorded with different detectors, which were previously refined independently to give two 3.0-Å maps (*39*), were combined. Initial attempts using RELION-3.0 improved the resolution moderately to 2.91 Å but led to loss of definition of the density for the inhibitor molecule, so the data merge was performed along with stricter filtering of poorer-quality particles and correction for higher-order aberrations with RELION 3.1 (*68*) (fig. S4).

Particles from the two datasets (*39*) were at slightly different pixel sizes, so the Falcon-3 (F3) data (1.0625 Å pixel^–1^) were reextracted with a box size of 504, and the K2 data (1.05 Å pixel^–1^) with a box size of 510 were rescaled to 504 pixels, both from micrographs motion-corrected using the RELION implementation of MotionCorr (*69*) with 1 × 1 patches and an amplitude contrast of 0.08. Images were CTF-corrected using exhaustive searches in CTFFIND 4.1.10 (*71*) using a maximum resolution for fitting of 5 Å. Both datasets were 3D refined using EMD-11424 (*39*) low-pass–filtered to 40 Å as an initial reference and further 2D classified without alignment to remove junk classes. The K2 dataset was polished using frames 1 to 21 (of 24), and the F3 dataset was polished using all frames. Particles with outlier pixel values (sigma value, >3) were removed. For the K2 dataset, particles with rlnMaxResolution >5.5 Å were removed, and for the F3 dataset, particles with rlnMaxResolution >3.2 Å were removed. Iterative rounds of CTF and 3D refinement were used to optimize the defocus, astigmatism, and B-factor on a per-particle basis, as well as the anisotropic magnification, beam tilt, trefoil, and fourth-order aberration estimations of the datasets (*68*). These processes were performed first on the two datasets independently and then on the combined dataset of 41,670 particles in two optics groups until no further improvements were observed. A final refinement was performed with Pad 1 and solvent-flattened FSCs. Then, the map was postprocessed using a mask created using the molmap command in UCSF Chimera v.1.13.1 (*74*) from a near-final PDB, low-pass–filtered to 15 Å and with a soft edge of 12 added by RELION MaskCreate. More than half the particles were from the K2 dataset, so the K2 detector MTF was used for MTF correction. The final resolution at FSC = 0.143 was 2.84 Å.

### Model building, refinement, and validation

A previously reported model for mouse complex I (PDB: 6ZR2) (*39*) was rigid-body fitted into the active-state map determined here using UCSF Chimera v.1.13.1, Curlew all-atom-refined using Coot v.0.9-pre (*76*), and then refined against the Phenix-autosharpened map by cycles of manual adjustment in Coot v.0.9-pre/v.0.9.2. Further cycles of Curlew all-atom refine and real space refinement in Phenix v.1.18.2-3874 (*75*) were performed, initially with a nonbonded weight of 300 and Ramachandran plot restraints set to Oldfield (favored) and Emsley8k (allowed and outlier). Densities for existing and additional ligands, lipids, and DDM molecules were identified with the Unmodelled blobs tool in Coot, and fatty acid tails were clipped where necessary using the delete tools in Coot and PyMOL. Water molecules were placed into distinct density peaks identified with the Find Waters function in Coot, with the search parameters set to 5.6 root mean square deviation for density peaks, with minimum and maximum distances to protein atoms of 2.4 and 3.4 Å, respectively. The waters identified were manually inspected to remove falsely placed and bulk solvent cases and to add additional waters that were missed in the search. Atom resolvability *Q* scores were measured using MapQ (*77*), and any outliers identified were corrected in Coot. The model was rechecked manually in Coot and real space refined in Phenix as outlined above but with a nonbonded weight of 100. The final model for the active-state map, without waters, was fitted into the piericidin-bound map using UCSF Chimera (*74*) and a piericidin A1 molecule manually fitted into its density identified in the map. Waters were added, and the model was refined and inspected out as described above. Model statistics (table S1) were produced using the Phenix implementations of MolProbity v.4.4 and EMRinger. Model-to-map FSC curves were generated using Phenix Comprehensive Validation Tools (cryo-EM), and the degree of directional resolution anisotropy calculated using the 3DFSC program suite (*78*).

### Structure analyses

The interior surfaces of the ubiquinone-binding, E-channel, and ND5 IMS-facing cavities were predicted using CASTp (*79*), which computes a protein surface topology from a PDB model, with the default 1.4-Å-radius probe. Hydrogen bonds were identified using the default hbonds command in UCSF ChimeraX (*80*), and Grotthuss-competent networks were identified using the contacts command, with the distanceOnly option set to 4 (to implement a center-to-center distance cutoff of 4 Å) and the restrict option used to limit interactions to protonatable O and N centers of Grotthuss-competent residues D, E, H, K, S, T, and Y and water molecules only.
