# Supplementary material for: Investigation of hydrated channels and proton pathways in a high-resolution cryo-EM structure of mammalian complex I
Source: Sci Adv. 2023 Aug 2;9(31):eadi1359. doi: 10.1126/sciadv.adi1359 (PMC10396290; doi:10.1126/sciadv.adi1359)
Supplement: Supplementary file 1 — Figs. S1 to S7 Tables S1 and S2 [file sciadv.adi1359_sm.pdf]

Science Advances
AAAS

Supplementary Materials for

Investigation of hydrated channels and proton pathways in a high-resolution cryo-EM structure of mammalian complex I

Daniel N. Grba *et al.*

Corresponding author: Judy Hirst, jh@mrc-mbu.cam.ac.uk

Sci. Adv. **9**, eadi1359 (2023)
DOI: 10.1126/sciadv.adi1359

This PDF file includes:

Figs. S1 to S7
Tables S1 and S2

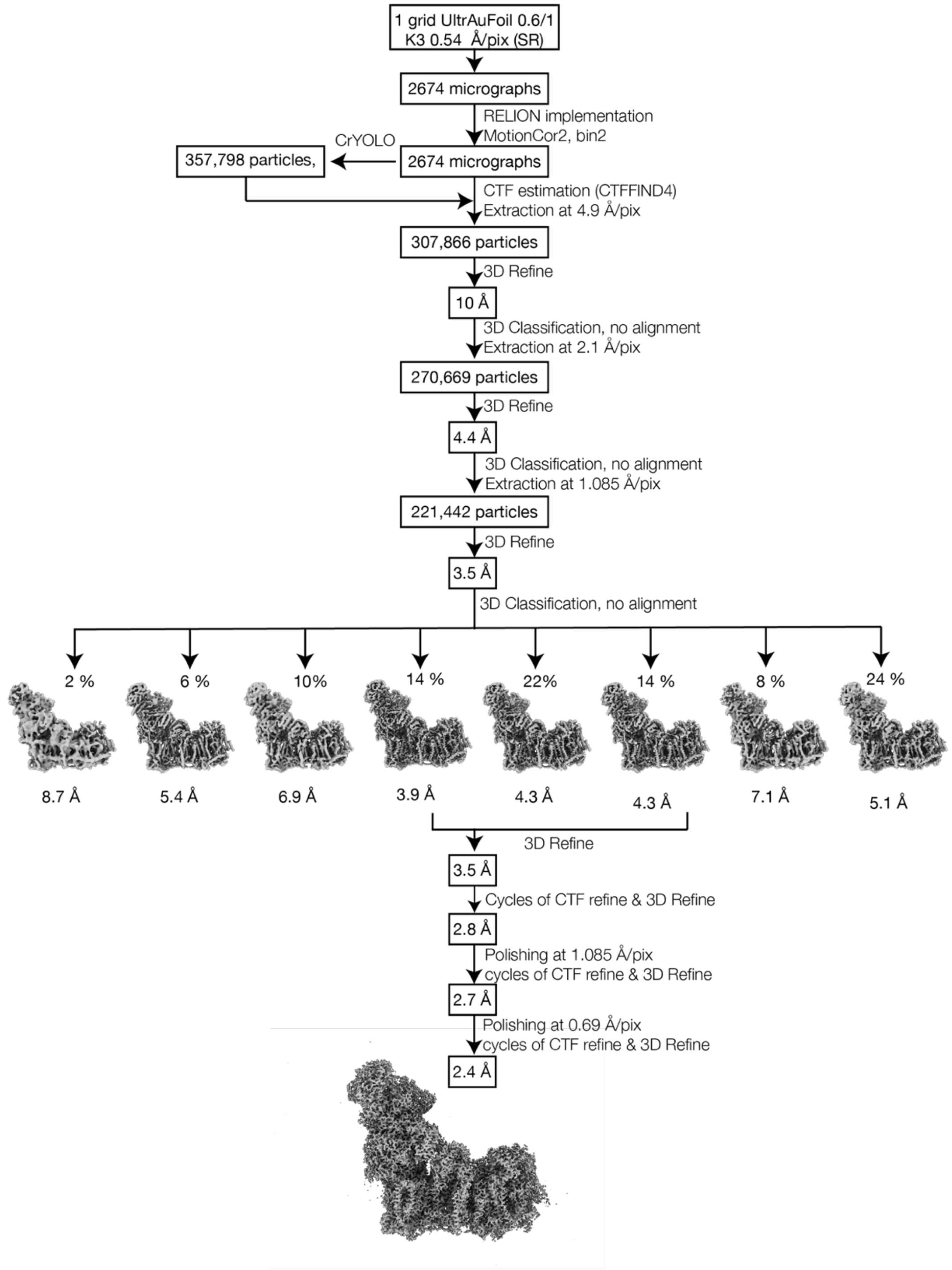

Supplementary Figure 1. High-resolution cryo-EM image processing scheme of mouse complex I. In the above image processing scheme, pre-processing steps such as automatic particle coordinate selection and CTF parameter estimation were carried out using crYOLO and CTFFIND-4.1, respectively (*71*, *72*). The rest of the image processing scheme was carried out using RELION-3.1 (*68*). The 6% class in the final round of 3D classification was observed to be in a conformation resembling the more globally open deactive state. All other classes either matched the active state or were too low resolution for further consideration. The three highest quality active state classes were combined and taken forward.

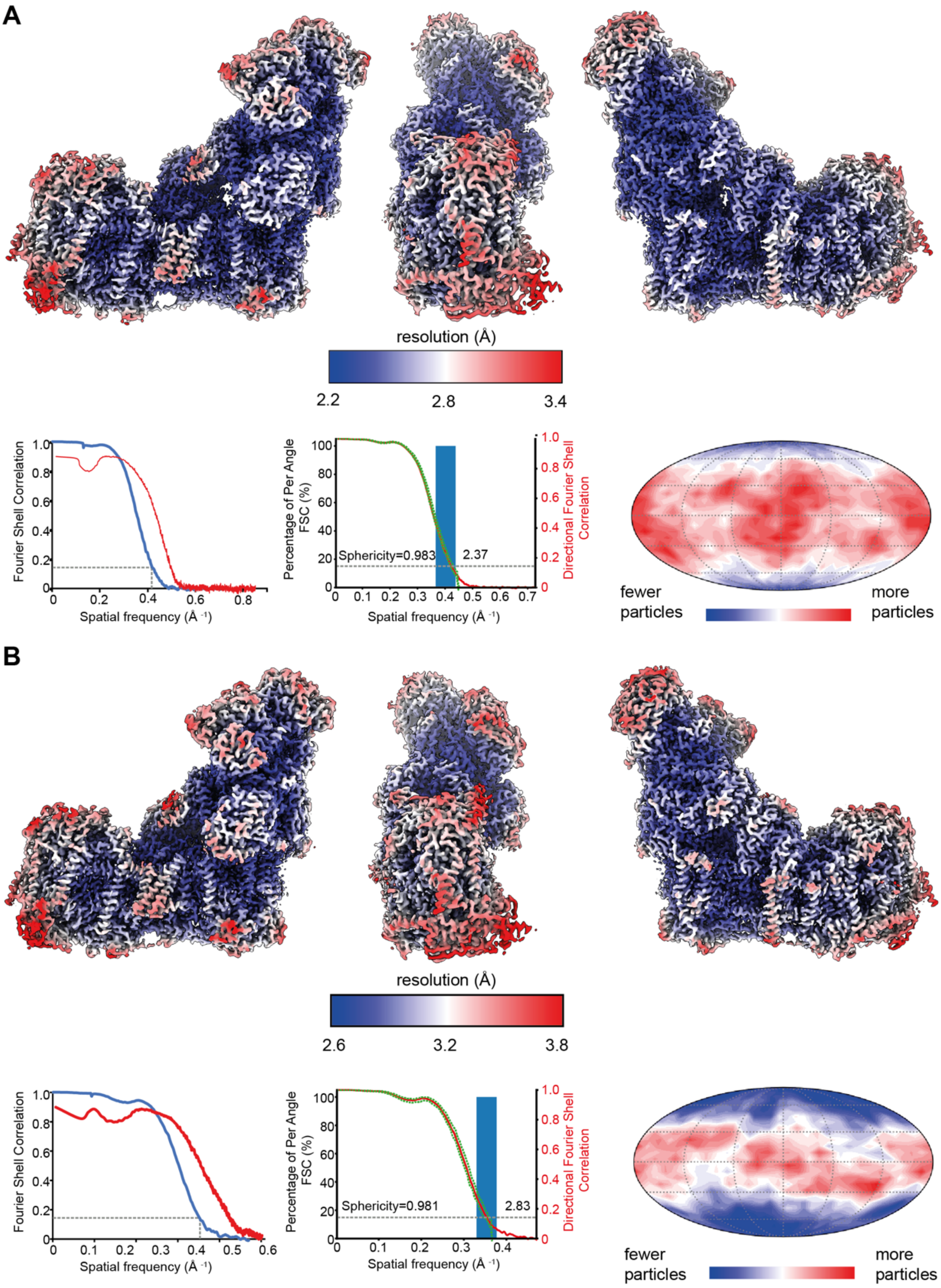

Supplementary Figure 2. Global and local resolution. (**A**) The active state of mouse complex I and (**B**) piericidin-inhibited mouse complex I. Each panel shows: the local resolution of the map; the Fourier shell correlation (FSC) between two independent half-maps, where the global resolution is estimated according to the gold-standard FSC at 0.143 (*73*), and the map-to-model FSC; 3D-FSC curves; and the Mollweide projection of the orientations. Local resolutions were estimated in RELION-3.1 using the RELION package and displayed using UCSF Chimera (*74*). Map-to-model FSC curves were produced using Mtriage in Phenix v.1.18.2. 3D-FSC curves were produced using the 3D-FSC server at the Salk institute (*78*).

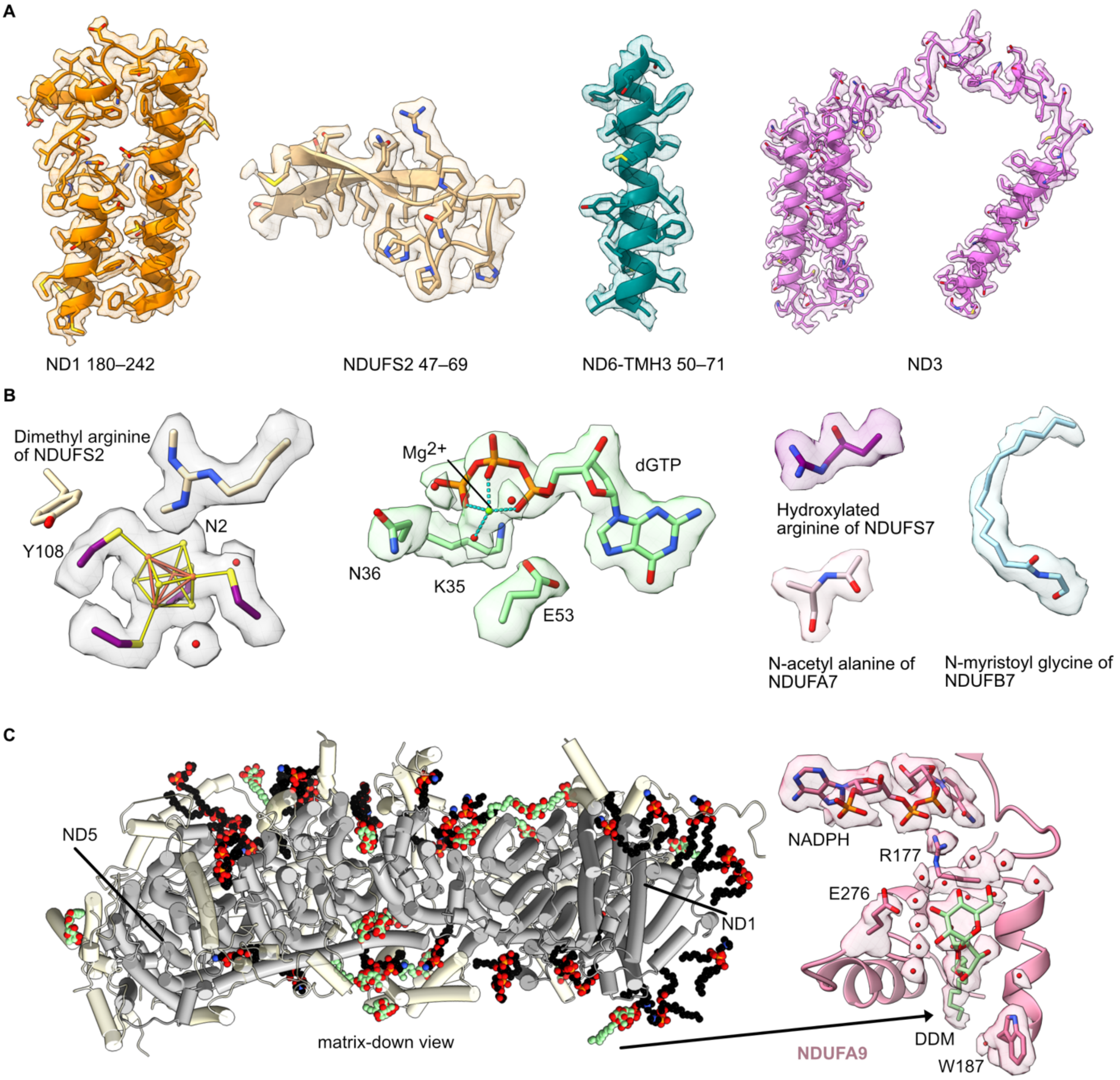

Supplementary Figure 3. Cryo-EM densities of key features. (**A**) Local conformations indicative of the active state. The cryo-EM densities of the auto-sharpened map display the ordered nature of the ND1-TMH5–6 and ND3-TMH1–2 loops, the NDUFS2-β1–β2 loop, and the α-helical structure of ND6-TMH3, which are all specific to the active (not the deactive) state. (**B**) The densities of ligands and post-translational modifications: the terminal FeS cluster N2 adjacent to dimethylated NDUFS2-Arg85; the dGTP–Mg^{2+} bound in NDUFA10 with surrounding cryo-EM densities; the γ-hydroxylation of NDUFS7-Arg87; the *N*-acetylation of NDUFA7-Ala1; and the *N*-myristoylation of NDUFB7-Gly1. (**C**) The modelled phospholipid (black) and DDM detergent (green) molecules shown from the matrix. The inset shows the cryo-EM density for the DDM modelled in an active-state binding pocket in NDUFA9 that has been reported previously to bind ubiquinone-10 (*12*) or phosphatidylethanolamine (*10*). All transparent surfaces depicting cryo-EM densities are shown at ChimeraX (*80*) map thresholds of 4.5, except for the *N*-myristoyl glycine and NDUFA9 inset which are 2.5 and 3, respectively. Densities are carved with a 1.5 Å distance. O atoms (including water molecules) are in red.

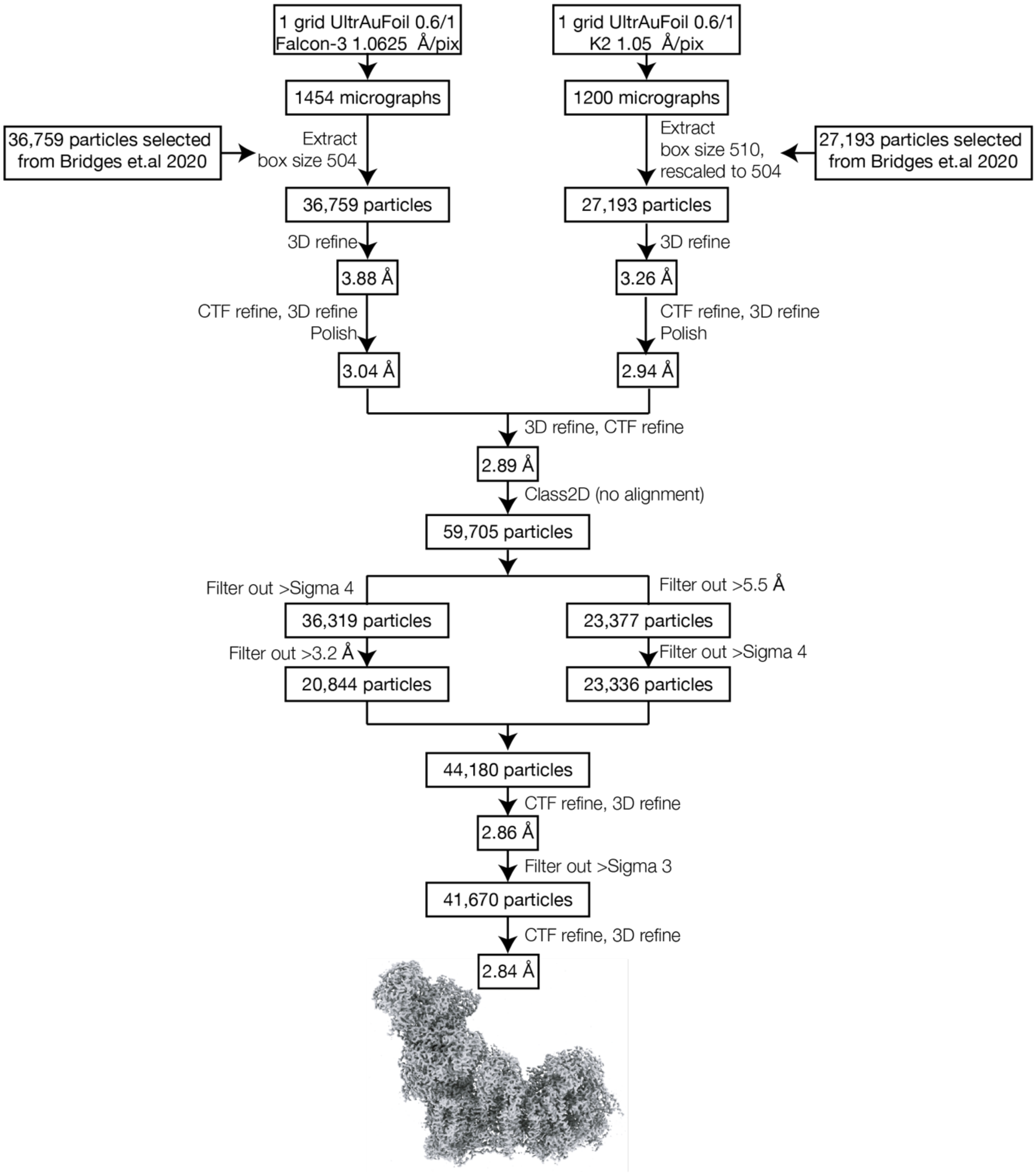

Supplementary Figure 4. Cryo-EM image processing scheme for piericidin-inhibited mouse complex I. Selected particles from two data collections (*39*) were further combined and processed using RELION-3.1.

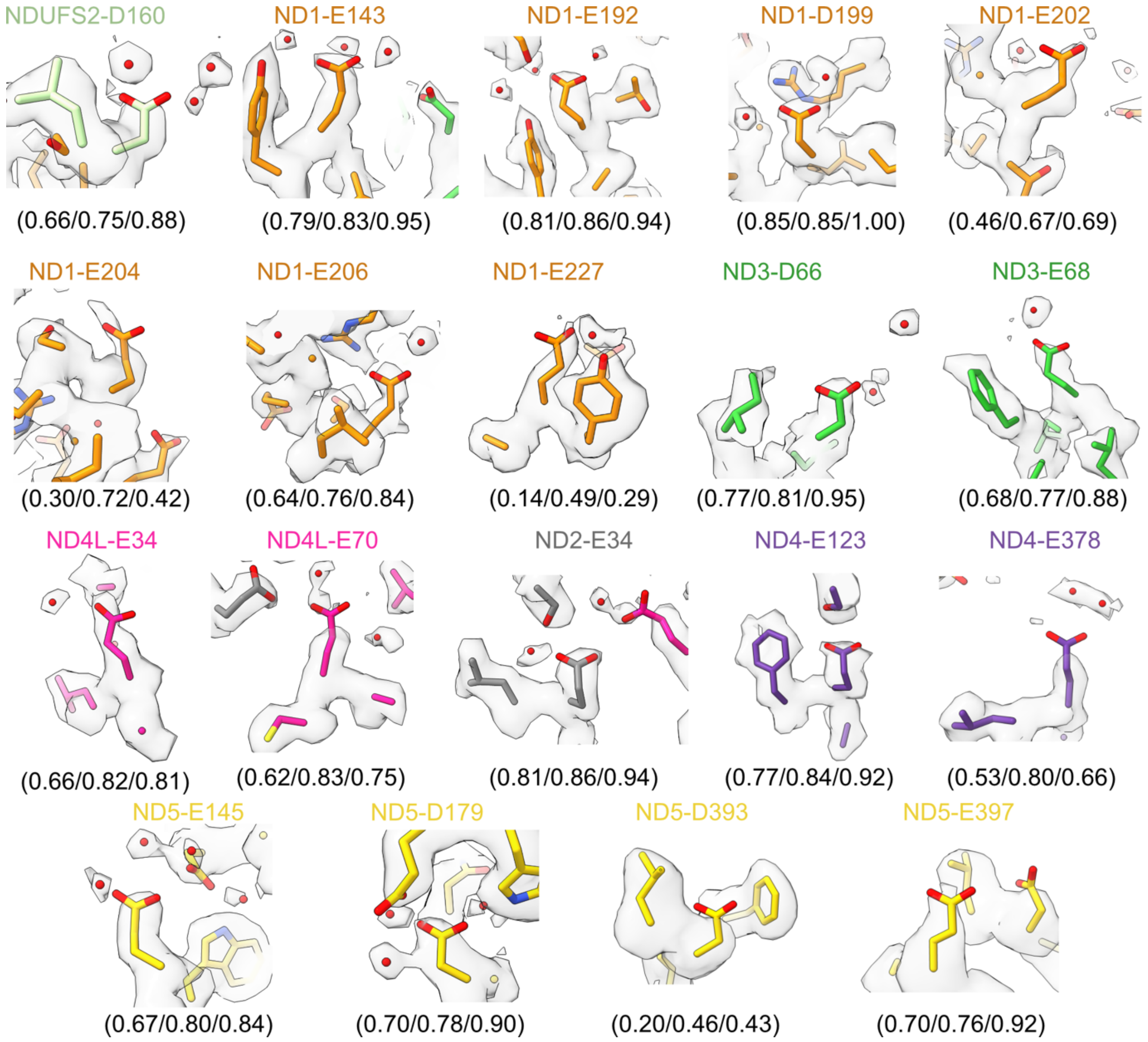

Supplementary Figure 5. The locally sharpened cryo-EM densities of key acidic residues and corresponding *Q*-scores. Panels show the cryo-EM outline at a 2 Å range from the displayed atoms (semi-transparent surface, ChimeraX map threshold 4.5) and each acidic residue is displayed with residues or water molecules within 3 Å distance of it. The values in parenthesis indicate the average oxygen *Q*-score (*77*), average residue *Q*-score, and the ratio of the two, respectively.

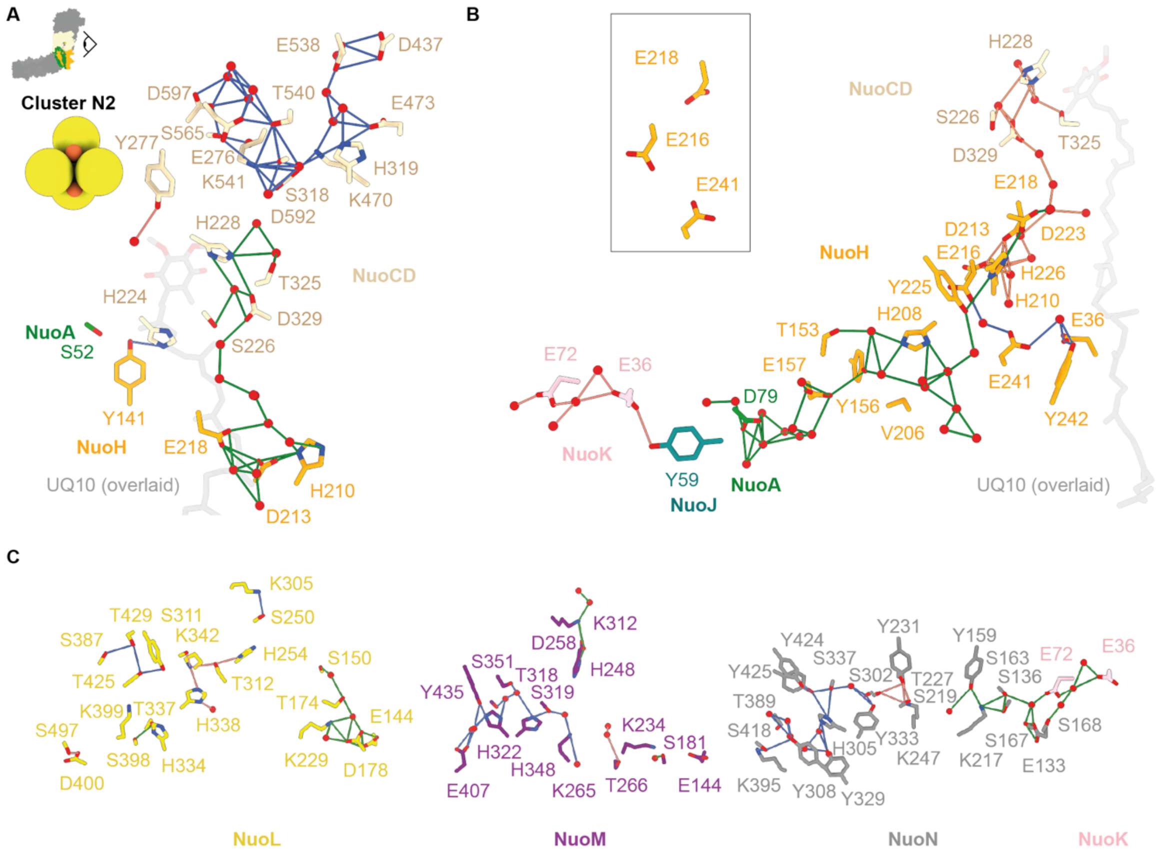

Supplementary Figure 6. The Grotthuss-competent networks of the ubiquinone-binding site and E-channel in the closed state of *E. coli* complex I at pH 6 (PDB: 7Z7S) (*16*). (**A**) Networks of Grotthuss-competent residues and water molecules connected to NuoCD-H224 (*Mm*-NDUFS2-H55), H228 (*Mm*H59), Y277 (*Mm*Y108) and K541:D592 (*Mm*K371:D422) in the active site for ubiquinone reduction. The upper-left icon indicates the viewpoint, with colors matching the residues labelled. (**B**) Grotthuss-competent networks in the E-channel. The inset shows the NuoH-E216 (*Mm*-ND1-E202) and E218 (*Mm*E204) sidechains in a downwards position, pointing towards E241 (*Mm*E227). Note that the network between NuoCD-H228 and ND1-D213 (*Mm*D199) is shown in both panels **A** and **B** and the UQ10 from PDB: 7QSK (*10*) is shown overlaid for reference. (**C**) Grotthuss-competent networks from NuoK-E36 (*Mm*-ND4L-E34) to NuoL (ND5) across the central hydrophilic axis. The residues are colored according to their subunit in Figure 2A, and the links within each network are colored differently to differentiate them. The panels and viewpoints match those shown in Figure 2C, 2D and 3A for the mouse enzyme.

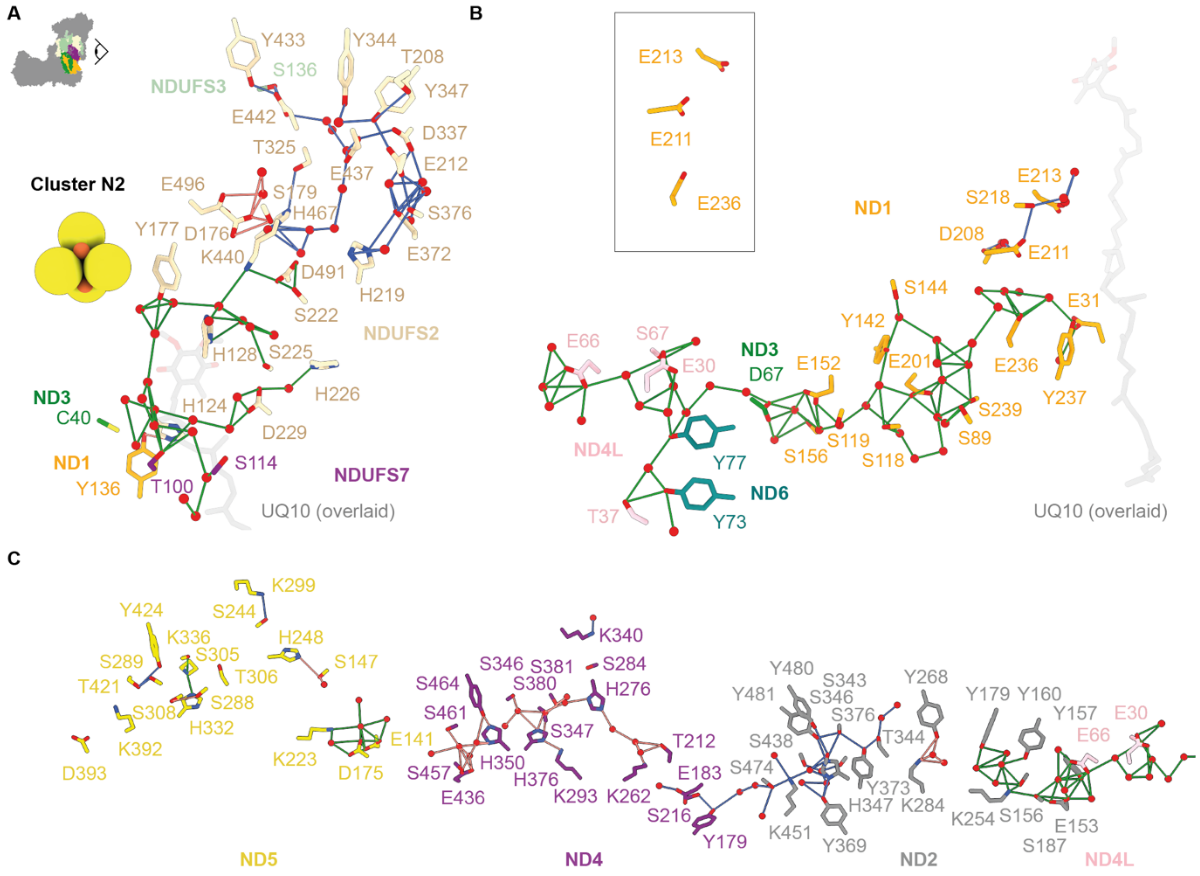

Supplementary Figure 7. The Grotthuss-competent networks of the ubiquinone-binding site and E-channel in the closed state ('state 2') of complex I from *C. thermophilum* (PDB: 7ZMB) (*29*)**.** (**A**) Networks of Grotthuss-competent residues and water molecules connected to NDUFS2-H124 (*Mm*-NDUFS2-H55), H128 (*Mm*H59), Y177 (*Mm*Y108) and K440:D491 (*Mm*K371:D422) in the active site for ubiquinone reduction. The upper-left icon indicates the viewpoint, with colors matching the residues labelled. (**B**) Grotthuss-competent networks in the E-channel. The inset shows the ND1-E211 (*Mm*E202) and E213 (*Mm*E204) sidechains in an intermediate position, relative to their positions in Figure 2D and Supplementary Figure 6. In panels **A** and **B** the UQ10 from PDB: 7QSK (*10*) is shown overlaid for reference. (**C**) Grotthuss-competent networks from ND4L-E30 (*Mm*E34) to NuoL (ND5) across the central hydrophilic axis. The residues are colored according to their subunit in Figure 2A, and the links within each network are colored differently to differentiate them. The panels and viewpoints match those shown in Figure 2C, 2D and 3A for the mouse enzyme.

Supplementary Table 1: Cryo-EM data collection, refinement and validation statistics for the active state of mouse complex I and mouse complex I with piericidin A bound.

	**Active state**<br>PDB: 8OM1<br>EMD-16965	**Piericidin A bound**<br>PDB: 8OLT<br>EMD-16962	
**Data collection and processing**			
Nominal magnification	81,000	130,000	47,600
Voltage (kV)	300	300	300
Electron exposure (e^{-} Å^{-2})	40	46	50
Targeted defocus range (μm)	-0.8 to -2.0	-2.2 to -3.8	-1.5 to -3.1
Calibrated pixel size (Å)	0.534 (super-res)	1.0625	1.05
Final sampling rate (Å pix^{-1})	0.6866	1.0625	
Symmetry imposed	*C*1	*C*1	
Initial particle images (no.)	307,866	63,962	
Final particle images (no.)	109,866	41,670	
Map resolution (Å) (FSC threshold)	2.39 (0.143)	2.84 (0.143)	
Map resolution range (Å)	2.176–5.892	2.61–5.76	
Map sharpening (RELION) *B* factor (Å^{2})	-17	-61	
**Refinement**			
Initial model used	6ZR2	6ZR2	
Model resolution (Å) (FSC threshold)	2.3 (0.5)	2.8 (0.5)	
Model composition			
Nonhydrogen atoms	71,256	68,075	
Protein residues	8,244	8,211	
Ligands	62	55	
Waters	2,945	280	
B factors mean (Å^{2})			
Protein	65.04	43.26	
Ligand	40.23	45.56	
Water	43.10	36.84	
RMS deviations			
Bond lengths (Å)	0.007	0.009	
Bond angles (°)	0.756	0.671	
Validation			
MolProbity score	1.24	1.61	
EMRinger score (PHENIX autosharpen)	5.67	4.32	
Clashscore	3.20	5.23	
Rotamer outliers (%)	0.87	0.38	
Cß outliers (%)	0.00	0.00	
Ramachandran plot			
Favored (%)	97.34	95.23	
Allowed (%)	2.61	4.70	
Outliers (%)	0.05	0.07	
Rama-Z (Ramachandran plot Z-score, RMSD)			
Whole	0.11 (0.09)	1.43 (0.09)	
Helix	0.26 (0.07)	1.05 (0.07)	
Sheet	0.14 (0.23)	0.65 (0.25)	
Loop	0.08 (0.11)	0.6 (0.11)	

Supplementary Table 2: Summary of the model for the active state of mouse complex I.

Subunit	Alternative names	Chain	Total residues	Modelled residues (%)	Modelled cofactors and modifications
NDUFV1	51 kDa, Nqo1, NuoF	F	444	9-438 (97.7)	FMN, 4Fe4S
NDUFV2	24 kDa, Nqo2, NuoE	E	217	4-217 (98.6)	2Fe2S
NDUFS1	75 kDa, Nqo3, NuoG	G	704	5-693 (97.9)	2Fe2S, 2 x 4Fe4S
NDUFS2	49 kDa, Nqo4, NuoC	D	430	1-430 (100)	Dimethyl-Arg85
NDUFS3	30 kDa, Nqo5, NuoC	C	228	7-214 (91.2)	
NDUFS7	PSST, Nqo6, NuoB	B	189	33-189 (83.1)	4Fe4S, Hydroxy-Cγ-Arg87
NDUFS8	TYKY, Nqo9, NuoI	I	178	1-178 (100)	2 x 4Fe4S
ND1	Nqo8, NuoH	H	318	1-318 (100)	N-formyl
ND2	Nqo14, NuoN	N	345	1-345 (100)	N-formyl
ND3	Nqo7, NuoA	A	115	1-115 (100)	N-formyl
ND4	Nqo13, NuoM	M	459	1-459 (100)	N-formyl
ND4L	Nqo11, NuoK	K	98	1-98 (100)	N-formyl
ND5	Nqo12, NuoL	L	607	1-607 (100)	N-formyl
ND6	Nqo10, NuoJ	J	172	1-172 (100)	N-formyl
NDUFV3	10 kDa	s	69	26-69 (63.8)	
NDUFS4	18 kDa	Q	133	8-133 (94.7)	
NDUFS5	15 kDa	e	105	1-105 (100)	2 x Cys-Cys
NDUFS6	13 kDa	R	96	1-95 (99.0)	Zn^{2+}
NDUFA1	MWFE	a	70	1-69 (98.6)	
NDUFA2	B8	S	98	13-98 (87.8)	
NDUFA3	B9	b	83	1-83 (100)	
NDUFA5	B13	V	115	2-115 (99.1)	
NDUFA6	B14	W	130	17-130 (87.7)	
NDUFA7	B14.5a	r	112	1-77, 89-112 (90.2)	N-acetyl
NDUFA8	PGIV	X	171	1-171 (100)	1 x Cys-Cys
NDUFA9	39 kDa	P	342	1-342 (100)	NADPH
NDUFA10	42 kDa	O	320	1-320 (100)	Mg^{2+}-dGTP
NDUFA11	B14.7	Y	143	1-142 (99.3)	2 x Cys-Cys
NDUFA12	B17.2	q	145	1-145 (100)	N-acetyl
NDUFA13	B16.6	Z	143	4-143 (97.9)	
NDUFAB1α	SDAPα	T	88	6-83 (88.6)	4’-phosphopantethine + 3-hydroxyundecanoate
NDUFAB1β	SDAPβ	U	88	1-88 (100)	
NDUFB1	MNLL	f	56	1-56 (100)	
NDUFB2	AGGG	j	72	4-69 (91.7)	
NDUFB3	B12	k	103	17-94 (75.7)	
NDUFB4	B15	m	128	3-128 (98.4)	
NDUFB5	SGDH	h	143	5-143 (97.2)	
NDUFB6	B17	i	127	1-36, 60-127 (81.9)	N-acetyl
NDUFB7	B18	o	136	1-119 (87.5)	N-myristoyl
NDUFB8	ASHI	l	157	1-157 (100)	
NDUFB9	B22	n	178	1-178 (100)	
NDUFB10	PDSW	p	175	3-172 (97.1)	
NDUFB11	ESSS	g	122	21-121 (82.8)	
NDUFC1	KFYI	c	49	1-49 (100)	
NDUFC2	B14.5b	d	120	1-120 (100)	
